# Chromosome Segregation and Cell Division Defects in *Escherichia coli* Recombination Mutants Exposed to Different DNA-Damaging Treatments

**DOI:** 10.3390/microorganisms11030701

**Published:** 2023-03-09

**Authors:** Ksenija Zahradka, Jelena Repar, Damir Đermić, Davor Zahradka

**Affiliations:** Laboratory for Molecular Microbiology, Division of Molecular Biology, Ruđer Bošković Institute, Bijenička 54, 10000 Zagreb, Croatia

**Keywords:** recombination pathways, DNA repair, double-strand breaks, single-strand gaps, RecBCD, RecFOR, RuvABC, RecG, chromosome segregation defects, stalled replication forks

## Abstract

Homologous recombination repairs potentially lethal DNA lesions such as double-strand DNA breaks (DSBs) and single-strand DNA gaps (SSGs). In *Escherichia coli*, DSB repair is initiated by the RecBCD enzyme that resects double-strand DNA ends and loads RecA recombinase to the emerging single-strand (ss) DNA tails. SSG repair is mediated by the RecFOR protein complex that loads RecA onto the ssDNA segment of gaped duplex. In both repair pathways, RecA catalyses reactions of homologous DNA pairing and strand exchange, while RuvABC complex and RecG helicase process recombination intermediates. In this work, we have characterised cytological changes in various recombination mutants of *E. coli* after three different DNA-damaging treatments: (i) expression of I-*Sce*I endonuclease, (ii) γ-irradiation, and (iii) UV-irradiation. All three treatments caused severe chromosome segregation defects and DNA-less cell formation in the *ruvABC, recG*, and *ruvABC recG* mutants. After I-*Sce*I expression and γ-irradiation, this phenotype was efficiently suppressed by the *recB* mutation, indicating that cytological defects result mostly from incomplete DSB repair. In UV-irradiated cells, the *recB* mutation abolished cytological defects of *recG* mutants and also partially suppressed the cytological defects of *ruvABC recG* mutants. However, neither *recB* nor *recO* mutation alone could suppress the cytological defects of UV-irradiated *ruvABC* mutants. The suppression was achieved only by simultaneous inactivation of the *recB* and *recO* genes. Cell survival and microscopic analysis suggest that chromosome segregation defects in UV-irradiated *ruvABC* mutants largely result from defective processing of stalled replication forks. The results of this study show that chromosome morphology is a valuable marker in genetic analyses of recombinational repair in *E. coli*.

## 1. Introduction

Homologous recombination (HR) is indispensable for accurate and efficient repair of potentially lethal genomic lesions, such as double-stranded DNA breaks (DSBs) and single-stranded DNA gaps (SSGs) [1,2,3,4]. In *Escherichia coli* and many other bacterial species, homologous recombination almost entirely depends on the RecA protein. This recombinase plays a key role during the central, synaptic stage of the recombination process that includes reactions of homologous DNA pairing and DNA strand exchange. The synaptic stage is prepared by binding RecA protein to single-stranded DNA (ssDNA), which is mediated by RecBCD and RecFOR protein complexes [3,5]. The mediator proteins play a crucial role by helping RecA to overcome the SSB protein during competition for ssDNA. Depending on the mediator complex involved in this process, two major recombination pathways, RecBCD and RecFOR, are distinguished in *E. coli* (reviewed in [1,3]). Polymerisation of RecA molecules on ssDNA in 5′-3′ direction gives rise to a nucleoprotein filament (termed RecA filament), which subsequently invades available DNA duplexes and searches for homology [5]. After pairing with homologous DNA, the invading RecA filament displaces one of the resident strands thus forming three-strand recombination intermediate called D-loop. During further reactions, D-loop is transformed into a four-strand intermediate named Holliday junction (HJ). Eventually, HJ is resolved into separate recombinant DNA molecules by the RuvABC protein complex that possesses both branch migration and endonuclease (resolvase) activities [6]. HJ may also be processed by an alternative, but only partially understood mechanism that involves the RecG helicase and possibly, an unknown nuclease activity [7,8,9].

DSBs represent the most severe form of DNA damage. They arise in the chromosome after treatment with various DNA-damaging agents (e.g., ionising radiation, UV light, bleomycin, nalidixic acid, etc.) or after induction of nucleolytic cleavage [4]. However, DSBs may also occur spontaneously during normal, unperturbed growth of cell cultures. Spontaneous DSBs result mainly from DNA replication accidents that occur when replication fork encounters a damaged DNA template or different obstacles on its path [10]. The RecBCD pathway is responsible for the majority of recombinational repair at DSBs in wild-type *E. coli* [1]. The RecBCD enzyme binds to the blunt or nearly blunt double-stranded DNA (dsDNA) ends, and then unwinds and simultaneously degrades both strands of the DNA duplex [11]. When the enzyme meets a regulatory octanucleotide sequence called Chi, its 3′-5′ exonuclease activity is silenced while its 5′-3′ exonuclease activity is increased. This Chi-dependent modification allows RecBCD to produce a long 3′-ending ssDNA tail [12]. The modified RecBCD also gains the ability to direct loading of the RecA protein onto newly produced ssDNA, thus promoting formation of the RecA filament [13,14].

In wild-type *E. coli*, the RecFOR pathway is used primarily for recombinational repair at SSGs [15], which arise in chromosomes when replication forks skip noncoding lesions (such as UV-induced photoproducts), or arise at stalled replication forks. UV light is a natural DNA-damaging agent that is frequently used in DNA repair studies, particularly those focused on SSG repair. The primary DNA lesions induced by UV are pyrimidine dimers that are subject to nucleotide excision repair (NER) [16]. However, the presence of pyrimidine dimers may alter DNA replication and lead to formation of secondary lesions such as SSGs and DSBs which require recombinational repair in order to be mended [17,18]. SSGs contain a region of ssDNA that is immediately available for RecA loading. Prior to RecA loading, the ssDNA region can be further enlarged by the action of RecQ helicase and several ssDNA exonucleases of different polarities [19]. However, the critical role in initiation of SSGs repair is played by RecFOR proteins that catalyse loading of RecA protein onto ssDNA thus enhancing the formation of RecA filaments within the SSGs [15]. 

The formation of DSBs in UV-irradiated bacterial cells is well documented, however, the underlying molecular mechanisms are quite complex and still not fully elucidated. DSBs in UV-irradiated cells may arise from: breakage of regressed replication forks [20,21], overlapping NER tracts during removal of closely spaced pyrimidine dimers located on opposite DNA strands [22,23], replication forks running into non-repaired SSGs [18], and cleavage of ssDNA within the SSGs [17,24,25]. Irrespective of their origin, the repair of DSBs in UV-irradiated cells proceeds mainly via RecBCD recombination pathway [21,25]. 

DNA damage not only interferes with the primary DNA functions (such as replication and transcription), but may also affect the global genome structure. Bacterial cells exposed to various DNA-damaging agents display pronounced changes in nucleoid morphology (such as nucleoid compaction and aggregation) and lack of normal nucleoid segregation [26,27]. These perturbations on the DNA level are usually accompanied by cell division defects including cell filamentation and aberrant divisions that produce anucleate cells [26,28,29,30]. The extent, duration and reversibility of the above cytological changes depend on the dose of the DNA-damaging agent applied as well as on the genetic background of the affected cells. 

Studies on *E. coli* and *Bacillus subtilis* have revealed that nucleoid compaction provoked by UV light or nalidixic acid largely depends on the functional RecA protein and formation of RecA filaments [26,27,31]. This suggests that ongoing recombinational repair may participate in chromosome reconfiguration after DNA damage. In line with this is finding that inactivation of the RecBCD enzyme (by a *recB* null mutation) suppresses nucleoid aggregation in *E. coli* cells after gamma (γ) irradiation [30]. In contrast, the *ruv*, *recG* and *ruv recG* mutants of *E. coli* which are deficient in resolution of recombination intermediates, show drastic nucleoid segregation and cell division defects after UV- or γ-irradiation [28,29,30]. Moreover, moderate chromosome segregation and cell division defects are present even in unirradiated, normally grown *ruv* and *ruv recG* mutants [29], and this phenotype is exacerbated in strains carrying additional recombination-related mutations [32]. This is compatible with finding that recombination intermediates (HJs) occasionally appear in exponentially growing cells as a result of the repair of spontaneous DNA damage [33]. 

In the present work, we have conducted a parallel analysis of different types of DNA damage (i.e., different DNA substrates), different mechanisms of their repair, and the consequent cytological effects. We have studied chromosome morphology and segregation after three different DNA-damaging treatments in *E. coli* strains carrying mutations in presynaptic (*recB*, *recO*) and postsynaptic (*ruvABC*, *recG*) recombination functions. The DNA-damaging treatments used were: (i) chromosome cleavage caused by expression of the yeast I-*Sce*I endonuclease, (ii) γ-irradiation, and (iii) UV-irradiation. The recombination mutants were also checked for their survival after particular DNA-damaging treatment. The results obtained served us to correlate typical cytological patterns of examined mutants with presumed recombinational repair defects associated with particular mutations. In this way, we systematised and supplemented the previous results of our and other research groups showing that chromosome morphology is a valuable marker in genetic analyses of recombinational repair pathways in *E. coli*. Our analysis enabled us to get novel insight into complex genetic interactions that occur during recombinational repair in *E. coli*, particularly in UV-irradiated cells that engage both major recombination repair pathways and which also use some of the recombination proteins for rescue of stalled replication forks. 

## 2. Material and Methods

### 2.1. Bacterial Strains, Growth Media and Conditions

All *E. coli* strains used for DNA repair and cytological experiments (Table S1 (Supplementary Materials)) were derivatives of strain MG1655 [34]. They were grown in LB liquid medium or on LB plates [35]. Cell growth in liquid medium was monitored on spectrophotometer, by measuring optical density at 600 nm (OD_600_). New strains were constructed by P1 transduction [35]. Mutations in the recombination genes (deletions or insertions) transferred by P1 transduction were associated with antibiotic resistance markers. Therefore, most transductants could be directly selected on LB plates supplemented with appropriate antibiotics: apramycin (Apra), 60 μg/mL; chloramphenicol (Cm), 15 μg/mL; kanamycin (Km), 25 μg/mL; tetracycline (Tc), 10 μg/mL; and trimethoprim (Tm), 100 μg/mL. In addition, transductants were checked for their UV sensitivity (UV^s^) phenotype (Table S1). The Δ*ruvABC::cam* mutation could not be directly introduced into the TRM387 background, which already contained the chloramphenicol resistance marker. In this case, the Δ*ruvABC::cam* mutation was co-tranduced with the close marker *eda-51*::Tn*10*. Co-transductants were first selected on plates with tetracycline and then screened for the Δ*ruvABC::cam* mutation using a UV sensitivity test.

### 2.2. Measurement of Bacterial Survival after Introduction of DSBs by I-SceI Endonuclease 

To study specifically DSB repair, we used an engineered *E. coli* strain TRM387 carrying both an arabinose-inducible cassette for expression of the yeast I-*Sce*I endonuclease, and an I-*Sce*I cleavage site cloned in the chromosomal *argE* locus [8]. During growth in the presence of arabinose, this strain expresses I-*Sce*I nuclease which then cleaves its target site and creates a DSB with two ends that have four nucleotides-long 3′ overhangs [8]. 

For the purpose of this study, TRM387 derivatives carrying mutations in various recombination genes were constructed by P1 transduction (Table S1). The DSB repair capacity of constructed strains was estimated essentially according to the procedure described previously [8]. Bacterial cultures were grown from a single colony in 20 mL of LB medium at 37 °C, and their growth was monitored by measuring OD_600_ (Figure S1). When the cultures reached the exponential phase (at OD_600_ of 0.2), each of them was divided into two equal parts. L-arabinose was added to one half of the culture (to induce expression of the I-*Sce*I endonuclease), and glucose to the other (to prevent I-*Sce*I expression). Both sugars were added to final concentrations of 0.2% (*w*/*v*). Incubation of the split cultures was continued at 37 °C for another 30 and 60 min. At these intervals, samples were taken for determination of cell viability. Cells from each culture were first washed by centrifugation and resuspension in phosphate buffer. Bacterial suspensions were then diluted in the same buffer to obtain a series of decimal dilutions. Five 10-μL aliquots of each dilution were spotted on LB medium plates. Colonies of viable cells were scored after 24 to 72 h of incubation at 37 °C. The number of colony-forming units (CFU) for each cell sample grown in the presence of arabinose was expressed relative to the CFU value of the corresponding control sample grown with glucose. The effect of arabinose-induced I-*Sce*I expression on cell growth was measured spectrophotometrically (Figure S1). 

### 2.3. Fluorescence Microscopy of Bacterial Cells after Induction of I-SceI Expression

Exponential cultures of TRM387 derivatives were grown as described above. After each culture reached an OD_600_ of 0.2, 2 mL samples were taken for microscopic analysis as a control. Arabinose was then added to the cultures (to a final concentration of 0.2% *w*/*v*) and incubation was continued for the next 60 min. Thereafter, the cells were centrifuged and transferred to arabinose-free medium in which they were grown for an additional 60 min. Finally, 2 mL cell samples were taken for microscopy. Control and arabinose-grown samples were centrifuged and fixed in 500 μL of 0.1% osmium tetroxide for 20 min. After fixation, the cells were centrifuged again and resuspended in 500 μL of 0.2M cacodylate buffer (pH 7.0). Their nucleoids were stained by adding the fluorescence dye 4′,6-diamidino-2-phenylindole (DAPI) to a final concentration of 1 μg/mL. After 20 min of staining, control cells and cells grown in arabinose were centrifuged and resuspended in 100 and 400 μL of cacodylate buffer, respectively. Glass slides for the microscopy were prepared as follows: 50 μL of 2% low gelling temperature agarose (Sigma-Aldrich, St. Louis, MO, USA) pre-warmed at 48 °C was spotted on each glass slide and covered with siliconised coverslip (dimensions 18 × 18 mm). After 2–3 min at room temperature, the coverslip was removed and 3 μL of prepared bacterial suspension was applied to a thin layer of solidified agarose. The agarose with cells was covered with a clean coverslip whose edges were then sealed with transparent nail polish. Cells were observed under Zeiss AxioVert 35 microscope adapted for combined phase contrast and fluorescence microscopy, and equipped with Zeiss Ph3 Plan-NEOFLUAR (100x/1.30 OIL) objective. Images were acquired using Pixera Pro150ES digital camera and Twain Viewfinder v.3.0 capture program. 

### 2.4. Measurement of Bacterial Survival after UV- and γ-Irradiation

Bacteria were grown from a single colony in LB medium at 37 °C and their growth was followed by measuring OD_600_ (Figure S2). For UV-irradiation, exponential bacterial cultures (at OD_600_ of 0.2) were diluted in phosphate buffer to obtain a series of decimal dilutions. Five 10-μL aliquots of each dilution were spotted on LB medium plates and exposed to different doses of UV light (wavelength, 254 nm) at a dose rate of 0.25 J m^−2^ s^−1^. The irradiation was carried out under the low pressure mercury germicidal lamp (Phillips), in conditions of subdued light to prevent photoreactivation. The dose rate was determined by VLX-3W radiometer (Bioblock). 

In γ-irradiation experiments, cells from exponential cultures were sedimented by centrifugation, resuspended in the same volume of cold phosphate buffer, and irradiated on ice with a ^60^Co γ-ray source at a dose rate of 3 Gy/s. The appropriate decimal dilutions of the cells in phosphate buffer were plated on LB agar plates. In both types of irradiation experiments, the colonies of survivors were counted after 24 to 72 h of incubation at 37 °C. The number of CFU obtained for each irradiated sample was expressed as a percentage of the CFU value of the respective non-irradiated control. 

### 2.5. Fluorescence Microscopy of UV- and γ-Irradiated Cells

Exponential bacterial cultures were prepared as described above. For UV-irradiation, cells from 6 mL of bacterial culture were harvested by centrifugation and resuspended in the same volume of ice cold phosphate buffer. The cell suspension was transferred into a glass Petri dish (diameter 10 cm) and irradiated with UV dose of 5 J m^−2^ (at dose rate of 0.25 J m^−2^ s^−1^) with swirling. Cells from 5 mL of irradiated suspension were precipitated by centrifugation, resuspended in 10 mL of LB medium and grown at 37 °C in water bath with shaking. Samples for microscopy were taken after 2 h of postirradiation incubation.

For γ-irradiation, 5 mL of cell suspension in phosphate buffer was transferred into a plastic tube and irradiated on ice with a ^60^Co γ-ray source at a dose of 100 Gy (dose rate 3 Gy/s). Irradiated cells were spun down by centrifugation, resuspended in 10 mL of LB, and grown as described above. Samples for microscopy were taken after 2 h of postirradiation incubation. In both types of irradiation experiments, the procedure of cell sampling and preparation of cells for microscopy was identical to that described above (see Section 2.3). After both γ- and UV-irradiation cell growth was monitored by measuring OD_600_ (Figure S2). 

### 2.6. Statistical Analyses

Statistical significance of differences in survival (after I-*Sce*I expression, gamma-radiation and UV radiation at 60 min, 200 Gy and 50 J/m^2^, respectively) was assessed by one-way ANOVA followed by Tukey HSD test. Statistical tests were carried out in R (version 4.2.0), with aov function for ANOVA (from the base package “stats”), and HSD.test (from the package “agricolae”, v.1.3-5) for Tukey HSD test with the grouping output. Pairwise *p*-values reported for the Tukey test are the *p*-values from the TukeyHSD function (from the base package stats) which reports the pairwise *p*-values adjusted for multiple testing. Differences in the production of filamentous and anucleate cells between different strains were assessed by the chi-square test of independence (function chisq.test from the base package “stats”). Post-hoc analysis was performed through pairwise chi-square tests between all the strains using function pairwiseNominalIndependence from the package “rcompanion” v.2.4.16. The pairwise *p*-values have been bonferroni-corrected for multiple testing. Due to the large number of comparisons and to keep the text readable, we abstained from citing all the *p*-values in the text. Instead, *p*-values are available in the Supplementary Materials. All the statements comparing different strains in the Results section are in agreement with the results of the statistical analysis.

## 3. Results

### 3.1. Effects of recB, recO, ruvABC and recG Mutations on Cell Survival and Morphology after Introduction of DSBs by I-SceI Endonuclease

A set of different recombination mutants was constructed in a genetic background carrying both an arabinose-inducible cassette for expression of the yeast I-*Sce*I endonuclease, and an I-*Sce*I cleavage site cloned in the chromosomal *argE* locus. The mutants were grown in the presence of arabinose in order to promote I-*Sce*I-mediated DSBs and samples were removed after 30 and 60 min to determine cell viability. As expected from previous studies [8,36], the recombination proficient (wild-type) strain showed only a moderate decrease in survival, whereas a much stronger effect was observed with the *recB* mutant which is deficient for DSB repair (Table 1). The *recO* mutant behaved essentially as the wild-type strain (Table 1, see also [36]). Additionally, the *recO* mutation had a negligible effect in the *recB* background (Table 1). These later findings are in line with the notion that the RecFOR pathway has only a marginal role in DSB repair. 

Inactivation of *ruvABC* and *recG* genes (which encode major postsynaptic functions) had different effects on cell survival after I-*Sce*I expression. The *ruvABC* mutant showed similar survival as the wild-type both after 30 min and 60 min of growth in arabinose (Table 1). This contrasts somewhat to the previous work that reported twofold lower survival of *ruvABC* mutant relative to wild-type after 30 min growth in arabinose [8]. In our experimental conditions, such a difference between *ruvABC* and wild-type cells was obtained only after 90 min of incubation in arabinose (not shown). In contrast, the *recG* mutant proved to be significantly more sensitive than the wild-type after 30 min and 60 min of I-*Sce*I expression (Table 1). The double *ruvABC recG* mutant showed the strongest drop of survival due to synergistic effects of the two mutations. Except for small discrepancy mentioned above, our results essentially corroborate those described previously [8].

We have next tested various combinations of mutations in presynaptic (*recB* and *recO*) and postsynaptic (*ruvABC* and *recG*) genes. In brief, all strains carrying *recB* mutation in combination with *ruvABC*, *recG*, and *ruvABC recG* mutations showed essentially the phenotype of the single *recB* mutant (Table 1). Notably, the *recB ruvABC recG* mutant showed 5- to 6-fold better survival than *ruvABC recG* mutant suggesting that inactivation of *recB* precludes toxic (abortive) recombination that occurs in *ruvABC recG* mutant. Such a conclusion was corroborated by the cytological analysis of the above strains (see further in the text). Taken together, these results are compatible with a view that RecBCD enzyme acts before RuvABC and RecG enzymes during DSB repair. As expected from the above results, inactivation of the *recO* gene had no additional effects in *ruvABC*, *recG*, and *ruvABC recG* mutants (Table 1). Also, as expected, the *recB recO* mutant and its *ruvABC*, *recG* and *ruvABC recG* derivatives showed similarly low survival (Table 1).

**Figure 1:**
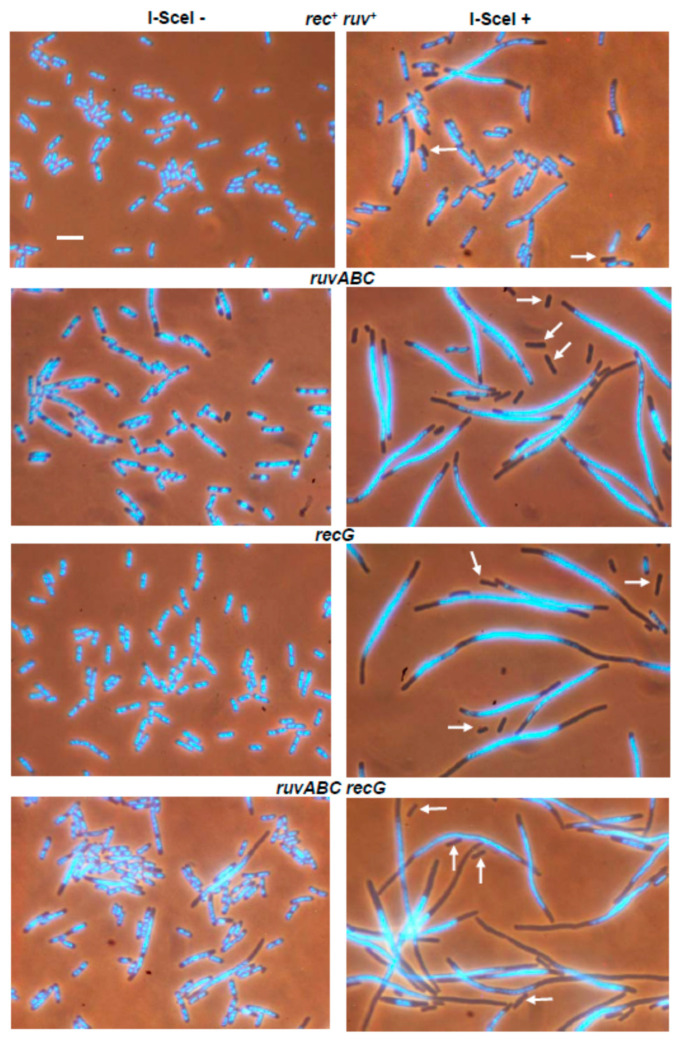
Changes in chromosome segregation and cell division induced by expression of I-*Sce*I endonuclease. Cell samples were taken before (**left** side of the panel) and after expression of I-*Sce*I (**right** side of the panel). Cells were fixed with osmium tetroxide, stained with DAPI and visualised using combined phase contrast and fluorescence microscopy. DAPI-stained chromosomes are seen as light blue structures within the dark outlines of cells. Arrows indicate anucleate cells. Bar, 5 μm.

The series of strains used in DSB repair essay was also examined for cytological changes associated with introduction of DSBs. For that purpose, the strains were first grown in the presence of arabinose for 60 min, and then transferred to arabinose-free medium and grown for additional 60 min. Upon completion of cultivation, the cells were examined under a fluorescence microscope. The wild-type population contained a significant number of filaments with large unsegregated nucleoids, and with DNA-free zones next to the cell poles (Figure 1, Table 2). Anucleate cells formed by separation from the ends of the filaments were also observed in the population. The described cytological changes were further enhanced in *ruvABC* and *recG* mutants, and especially in double *ruvABC recG* mutants. All these strains showed pronounced nucleoid aggregation and produced numerous anucleate cells (Figure 1 and Figure S3, Table 2).

**Figure 2:**
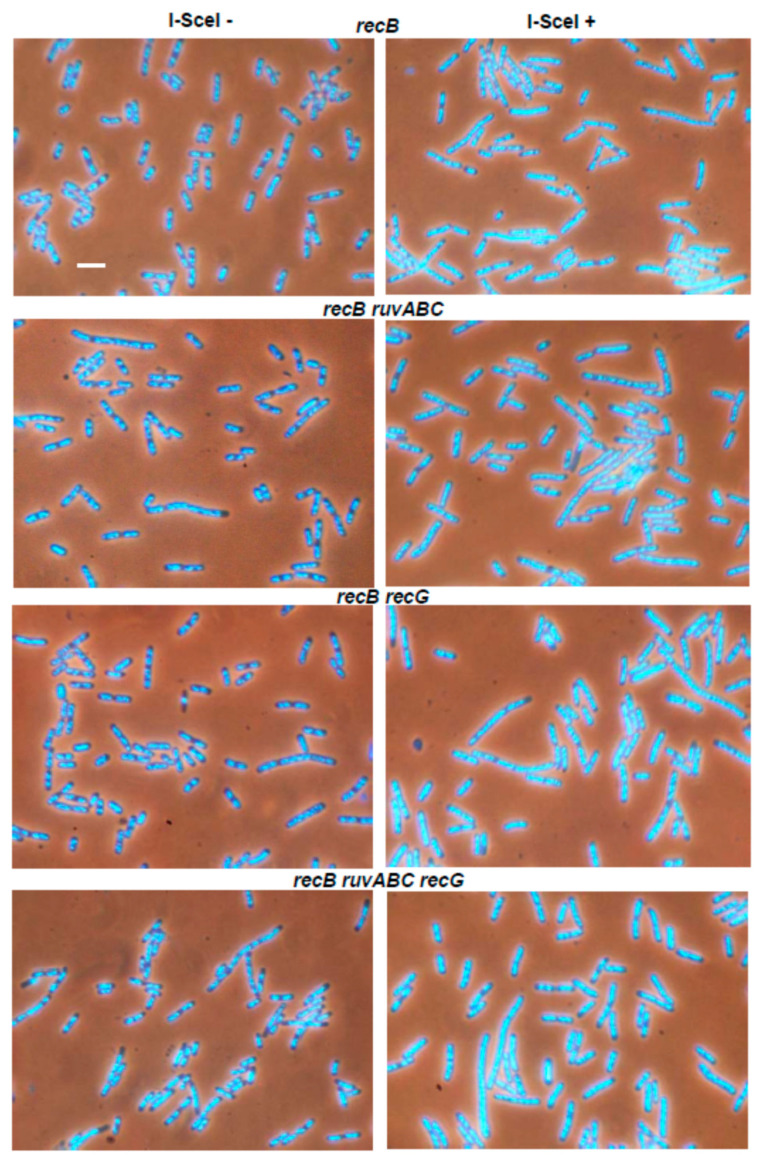
The *recB* mutation suppresses I-*Sce*I-induced cytological defects in *ruvABC* and/or *recG* mutants. Bar, 5 μm.

In contrast to the strains mentioned above, the *recB* mutant displayed less pronounced cytological changes after I-*Sce*I expression. This mutant produced fewer filaments than the wild-type strain (Table 2). The filaments contained DNA that was evenly dispersed throughout the cell volume, leaving no DNA-free zones in the cells (Figure 2). 

Consistent with this, no anucleate cells were observed in *recB* mutant culture (Table 2).

The *recB ruvABC*, *recB recG*, and *recB ruvABC recG* mutants were much more similar to the single *recB* mutants than the corresponding *ruvABC, recG* or *ruvABC recG* mutants (Figure 2, Table 2). In other words, the *recB* mutation ameliorated cytological defects associated with *ruvABC* and/or *recG* mutations.

**Figure 3:**
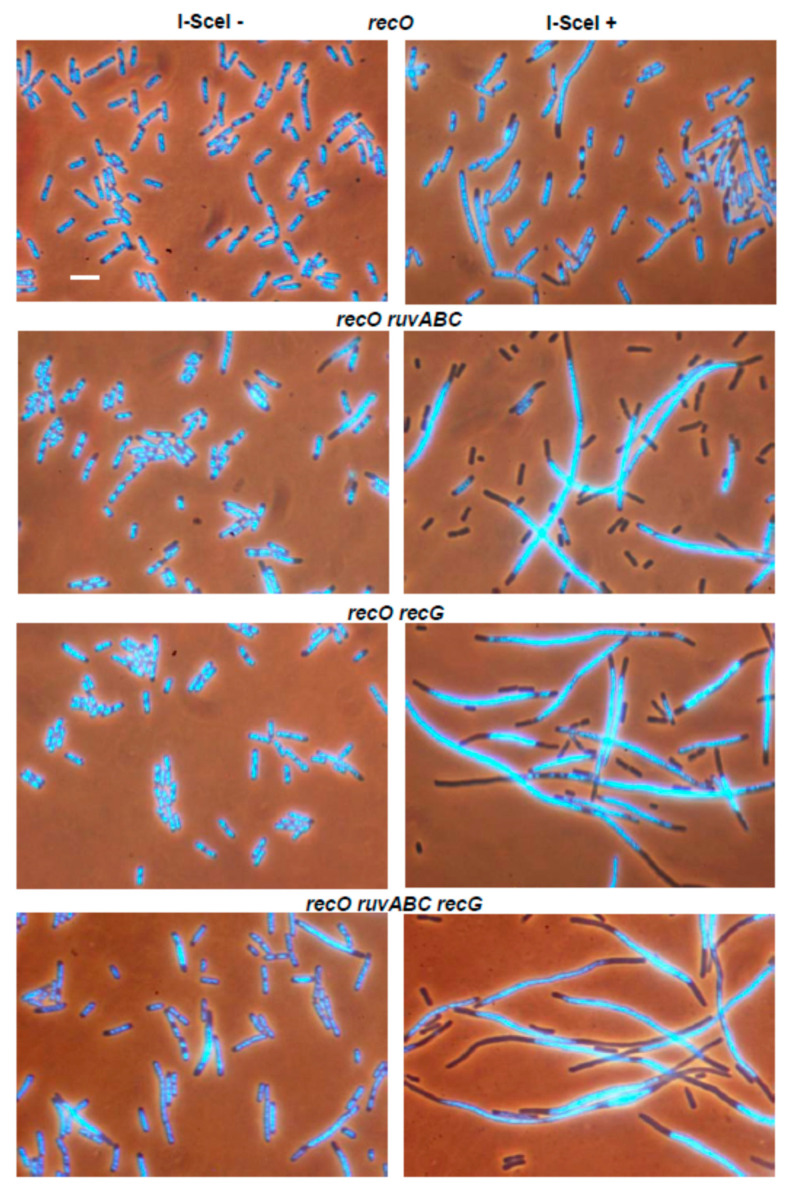
The *recO* mutation does not influence I-*Sce*I-induced cytological defects in *ruvABC* and/or *recG* mutants. Bar, 5 μm.

**Figure 4:**
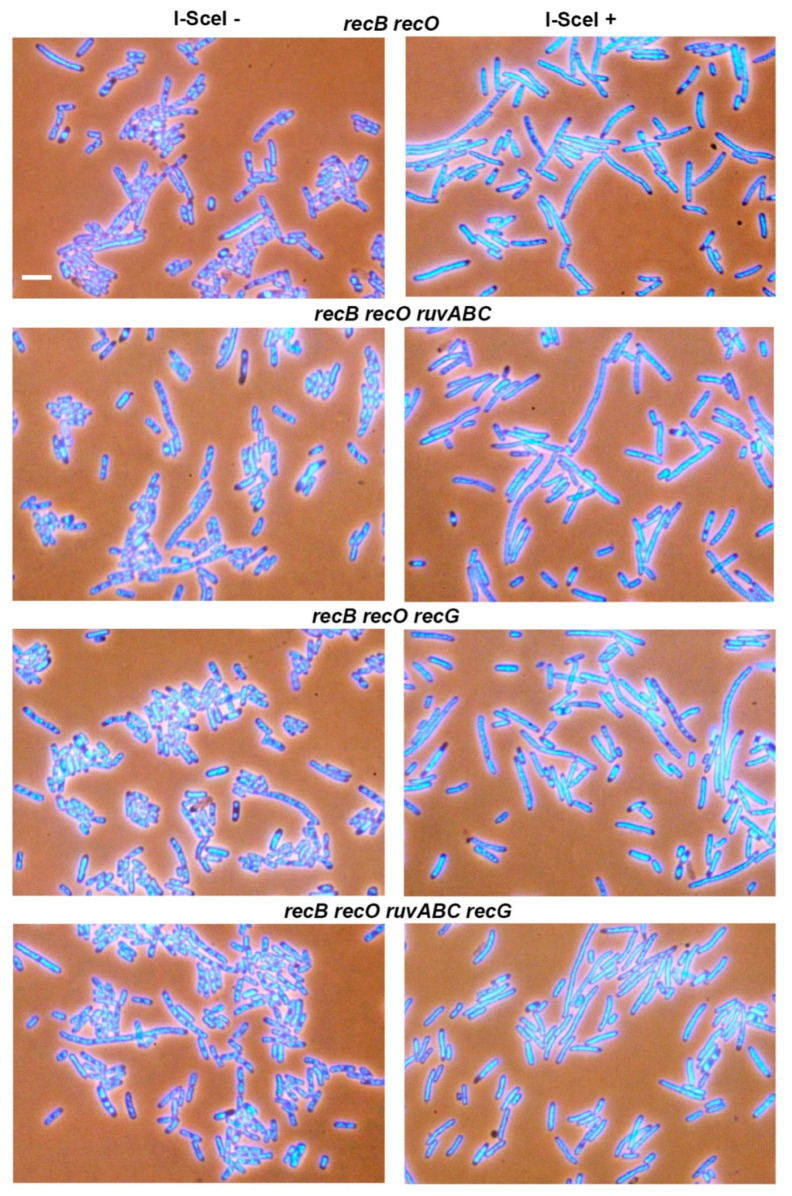
Effects of *recB recO* mutations on I-*Sce*I-induced cytological defects in wild-type, *ruvABC* and/or *recG* backgrounds. Bar, 5 μm.

In contrast to the effects of the *recB* mutation, inactivation of the *recO* gene had no strong cytological effect in either wild-type or in the *ruvABC*, *recG*, and *ruvABC recG* backgrounds (Figure 3, Table 2). As expected, the *recB recO* mutant and its *ruvABC, recG* and *ruvABC recG* derivatives showed a similar cytological pattern (Figure 4, Table 2). Additionally, this pattern was similar to that observed in the *recB* mutant, with or without additional *ruvABC*, *recG* and *ruvABC recG* mutations (compare Figure 2 and Figure 4, Table 2). Taken together, these results strongly suggest that nucleoid aggregation and other cytological defects caused by I-*Sce*I expression in *ruvABC*, *recG*, and *ruvABC recG* mutants result from incomplete DSB repair, i.e., from accumulation of unresolved recombination intermediates formed on the RecBCD recombination pathway.

### 3.2. Effects of recB, recO, ruvABC and recG Mutations on Cell Survival and Morphology after Exposure to γ-Irradiation

**Figure 5:**
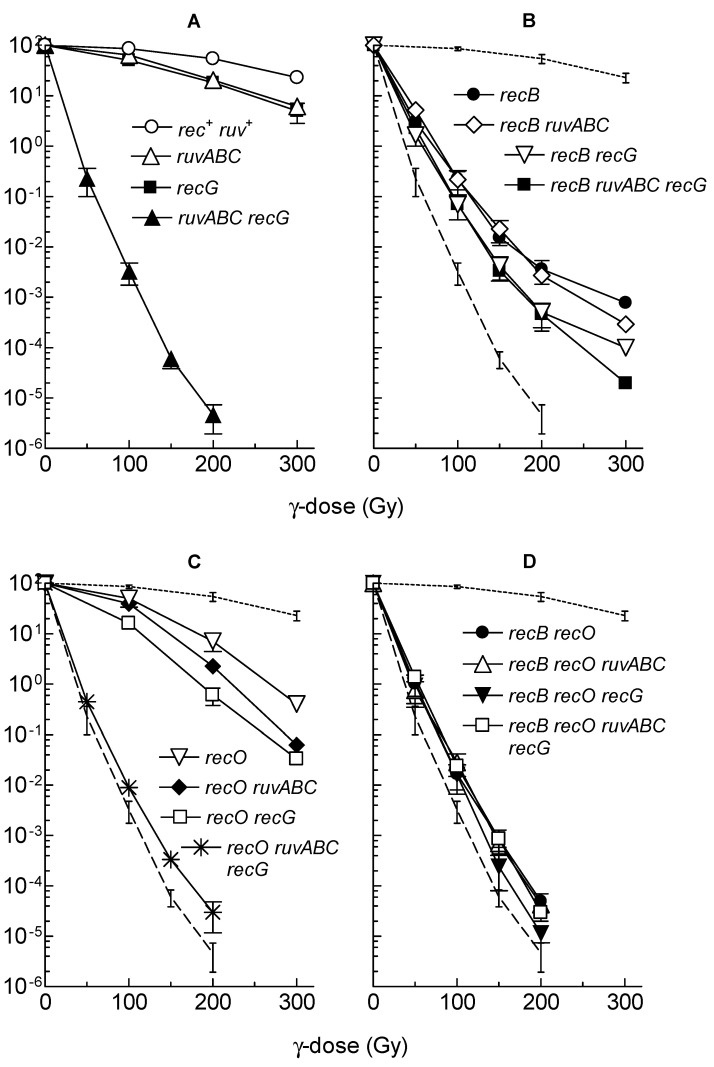
Survival of different *E. coli* mutants after γ-irradiation. The data for each strain are averages of results from at least three independent experiments, with error bars representing standard deviations. The strains used are: (**A**) *rec^+^ ruv^+^* (LMM2629), *ruvABC* (LMM3188), *recG* (LMM3196), *ruvABC recG* (LMM3610); (**B**) *recB* (LMM3183), *recB ruvABC* (LMM3625), *recB recG* (LMM3626), *recB ruvABC recG* (LMM4134); (**C**) *recO* (LMM3545), *recO ruvABC* (LMM3600), *recO recG* (LMM3601), *recO ruvABC recG* (LMM3604); (**D**) *recB recO* (LMM3599), *recB recO ruvABC* (LMM3602), *recB recO recG* (LMM3603), *recB recO ruvABC recG* (LMM3605). Dotted lines and dashed lines on panels (**B**–**D**) represent survival of the wild-type (*rec^+^ ruv^+^*) and the *ruvABC recG* mutant, respectively. Statistical analysis is provided in Tables S5 and S6.

Gamma irradiation is an agent that induces a variety of lesions to DNA. However, DSBs are generally considered to be responsible for most of the lethal effects associated with γ-irradiation [16]. To further study the role of the two major recombination pathways in DSB repair, we examined the effects of *recB*, *recO*, *ruvABC* and *recG* mutations on cell survival after exposure to different doses of γ-irradiation. The mutants used for this experiment were constructed in the genetic background of the strain MG1655 (Table S1). The radiation doses applied only slightly affected the survival of the wild-type strain (Figure 5A), whereas they caused a strong decrease of survival of the *recB* mutant (Figure 5B). Consistent with previous results [8], survival curves for individual *ruvABC* and *recG* mutants show similar modestly reduced survival, while the combined *ruvABC recG* mutant showed a dramatic reduction in cell survival (Figure 5A). The combination of *recB* and *ruvABC* mutations resulted in a phenotype quite similar to that of the single *recB* mutant, whereas *recB* and *recG* mutations produced a moderate synergistic effect (Figure 5B). The triple *recB ruvABC recG* mutant was basically as sensitive as the *recB recG* mutant. Interestingly, the *recB ruvABC recG* mutant survived γ-irradiation significantly better than the *ruvABC recG* strain (Figure 5A,B), reminiscent of the situation observed after induction of I-*Sce*I gene (see Table 1). 

Inactivation of the *recO* gene significantly reduced the survival of γ-irradiated cells (Figure 5C). It also produced a significant synergistic effect in combination with the *recB* mutation (Figure 5D). These results are in accord with previous studies showing that RecFOR pathway contributes to DNA repair after γ-irradiation, although its contribution is modest compared to that of the RecBCD pathway [37,38,39]. Combining the *recO* mutation with *ruvABC* or *recG* mutations resulted in a further moderate reduction in survival when compared to the single *ruvABC* and *recG* mutations (Figure 5C). However, the *recO* mutation did not result in the further reduction in survival of the *ruvABC recG* mutant (compare A and C in Figure 5). As expected, the *recB recO* double mutant and its *ruvABC, recG* or *ruvABC recG* derivatives had very similar survival curves (Figure 5D). The collective results described above suggest that RuvABC complex works predominantly on the RecBCD pathway, whereas the RecG protein works on both recombination pathways during DNA repair after γ-irradiation. 

**Figure 6:**
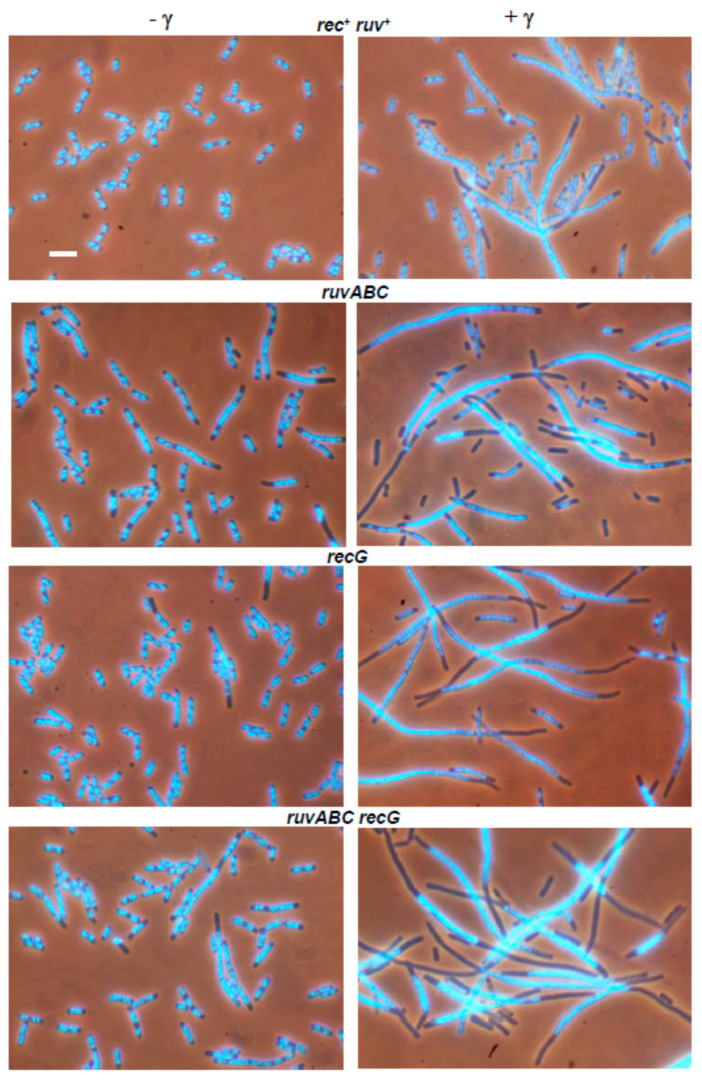
Chromosome segregation and cell division defects in wild-type, *ruvABC*, *recG* and *ruvABC recG* mutants after exposure to γ-irradiation. Exponentially growing cells, either non-irradiated (**left** side of the panel) or irradiated with 100 Gy of γ-irradiation and then grown for two hours (**right** side of the panel), were fixed with osmium tetroxide, stained with DAPI and observed under a fluorescence microscope. Bar, 5 μm.

Microscopic analysis of the wild-type strain after γ-irradiation showed moderate chromosome aggregation, together with cell filamentation and production of anucleate cells (Figure 6, Table 3), which is consistent with our previous results [30]. All cytological defects were more severe in *ruvABC, recG,* and *ruvABC recG* mutants (Figure 6). In particular, *ruvABC* and *ruvABC recG* mutants showed an extreme production of anucleate cells (Table 3) which was caused by frequent cell divisions in the absence of chromosome segregation. Chromosome segregation defect was most obvious in the *ruvABC recG* filaments, which had large nucleoids and long DNA-free regions (Figure 6).

**Figure 7:**
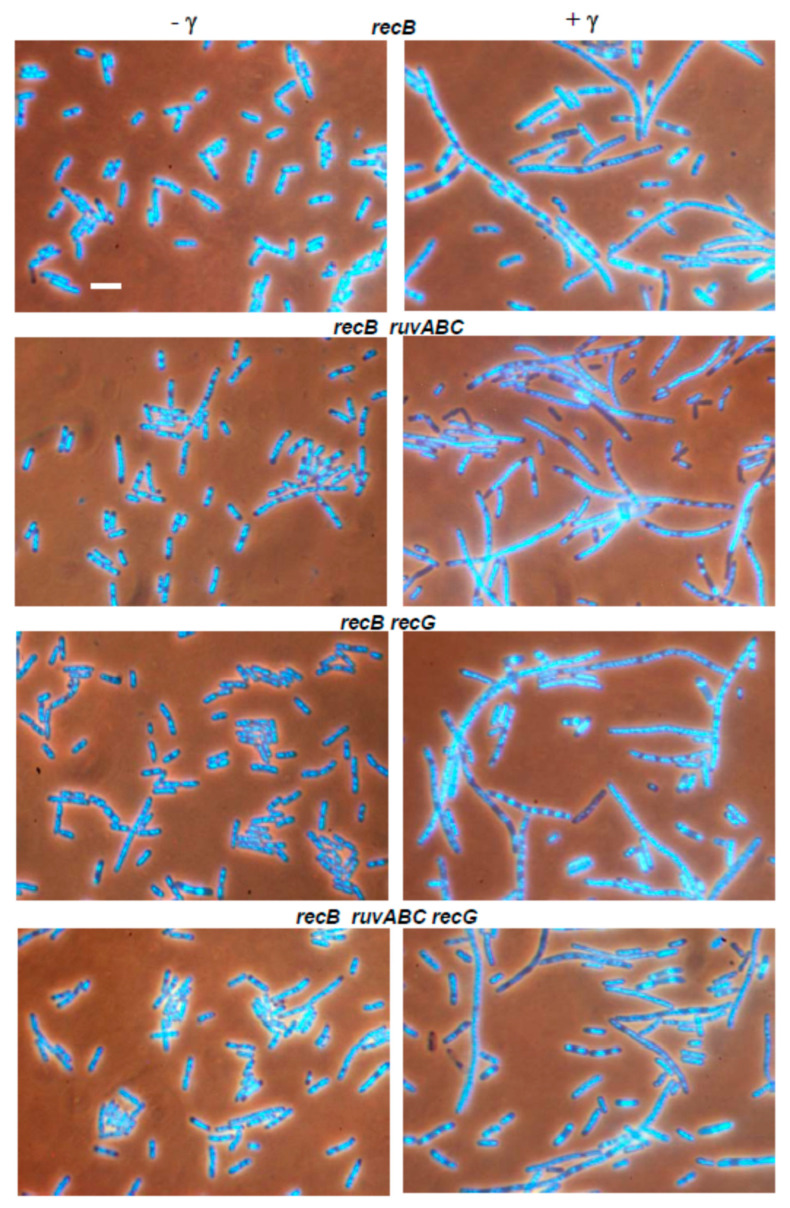
Suppressive effect of a *recB* mutation on cytological defects in γ-irradiated wild-type, *ruvABC*, *recG* and *ruvABC recG* strains. Bar, 5 μm.

As observed previously [30], the γ-irradiated *recB* mutant showed seemingly healthier morphology than the wild-type. Although some *recB* cells were filamentous (Figure 7), their DNA seemed to be on average less aggregated than in the wild-type cells (compare Figure 6 and Figure 7). γ-irradiated *recB* mutant did not produce DNA-less cells (Table 3). Furthermore, the *recB* mutation suppressed extreme chromosome aggregation in *ruvABC, recG,* and *ruvABC recG* mutants (Figure 6 and Figure 7). Interestingly, the *recB recG* and *recB ruvABC recG* mutants were morphologically almost identical to the *recB* mutant, while *recB ruvABC* mutant still displayed residual *ruvABC* phenotype that was particularly evident in a significant number of DNA-less cells (Table 3). Such a phenotype of the *recB ruvABC* mutant was somewhat surprising given that this mutant had γ-survival curve similar to that of the *recB* single mutant (Figure 5B, Table S5). Therefore, it is obvious that chromosome morphology and segregation are very sensitive to the lack of RuvABC function, so that microscopic examination can reveal a minor component of the *ruvABC* phenotype that remains unnoticed when measuring the survival of irradiated cells.

We drew the following conclusions from the above results. First, cytological defects of the γ-irradiated *recG* mutant are associated with a defective postsynaptic phase of RecBCD-mediated DSB repair. Second, most of the cytological defects of the γ-irradiated *ruvABC* mutant are associated with the RecBCD pathway, while the remainder of the defects are independent of RecBCD and occur in the presence of RecG function.

**Figure 8:**
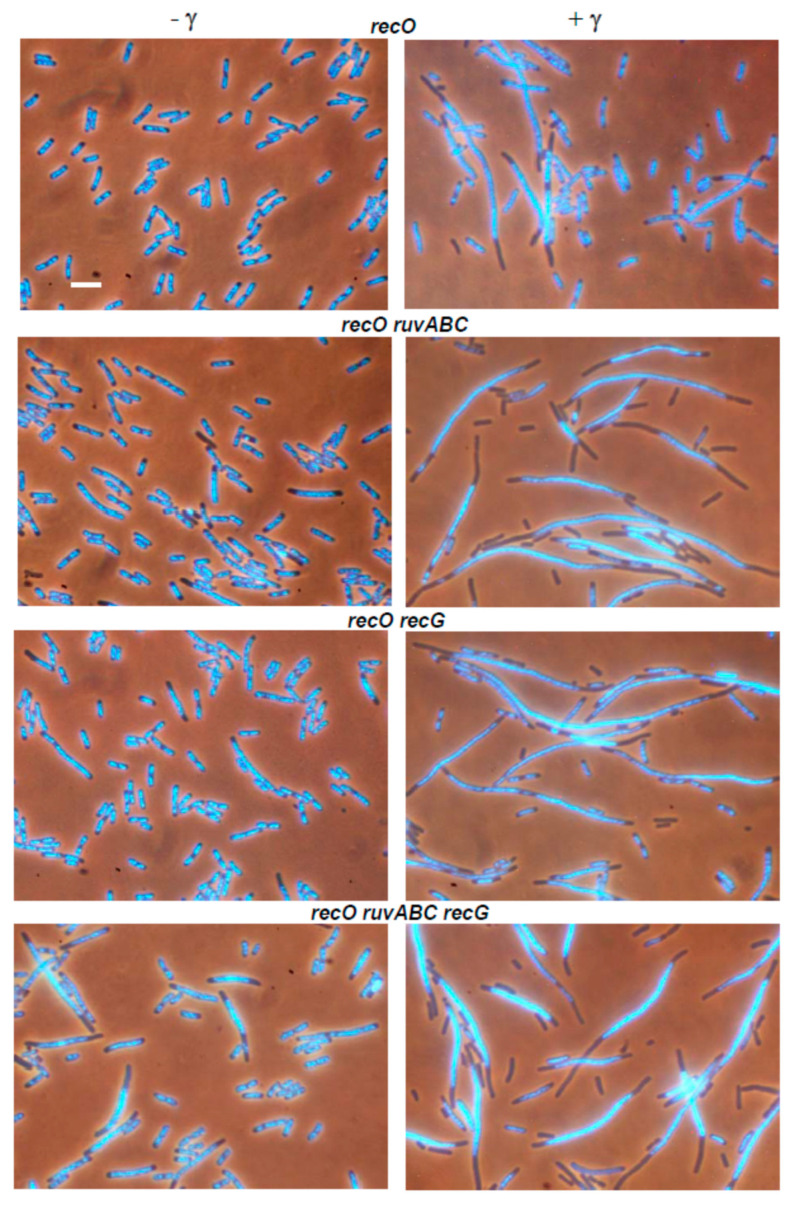
The *recO* mutation does not suppress γ-radiation-induced cytological defects in *ruvABC* and/or *recG* mutants. Bar, 5 μm.

**Figure 9:**
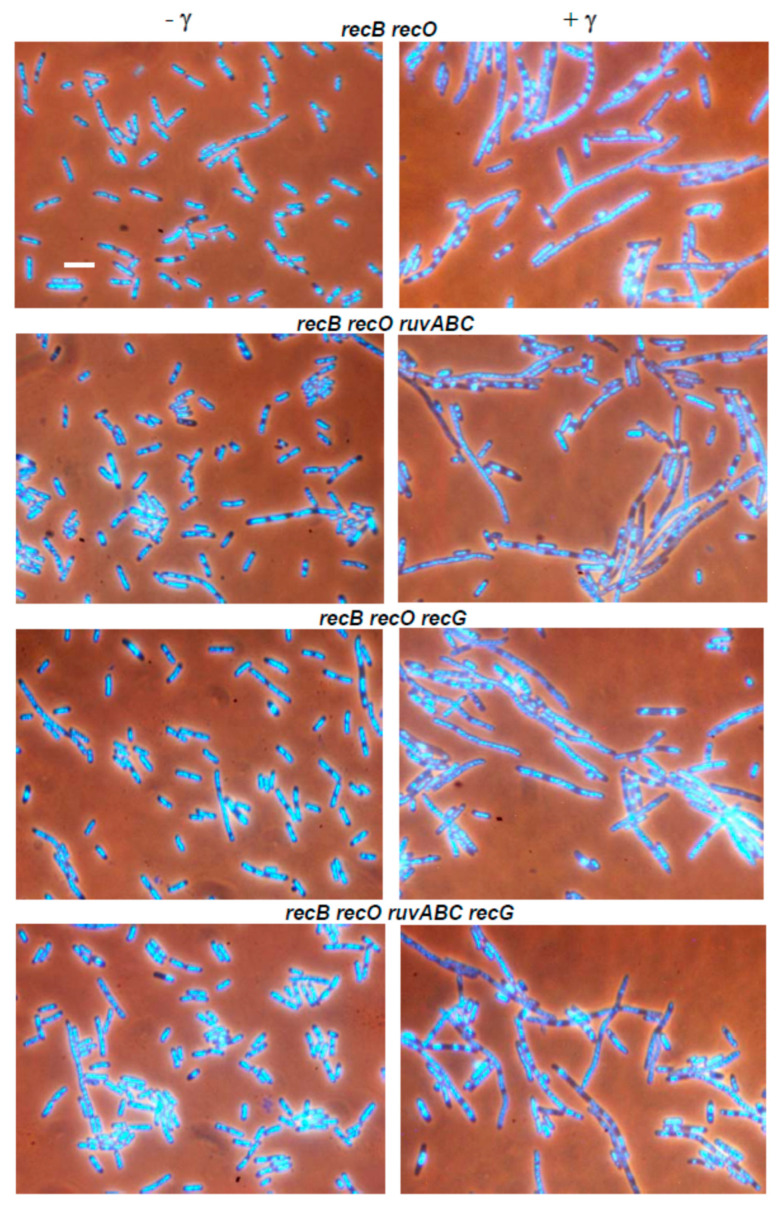
The combination of *recB* and *recO* mutations is required for complete suppression of chromosome segregation defects in γ-irradiated *ruvABC* mutants. Bar, 5 μm.

We also examined the cytological effects of γ-irradiation in strains carrying the *recO* mutation, either alone or in combination with other mutations of interest. Compared to the wild-type strain, the *recO* single mutant showed slightly stronger filamentation, nucleoid aggregation, and DNA-less cell production (Figure 8, Table 3). Introduction of the *recO* mutation into the *ruvABC* strain did not produce significant cytological effects when compared to the single *ruvABC* mutation (Figure 8, Table 3). The introduction of the *recO* mutation into the *recG* and *ruvABC recG* strains also did not produce strong cytological effects. Finally, we checked the postirradiation morphology of the double *recB recO* mutant and its derivatives carrying additional *ruvABC, recG,* or *ruvABC recG* mutations. All tested mutants showed essentially the same phenotype characterised by the absence of nucleoid aggregation and anucleate cells (Figure 9, Table 3). From these results, we infer that postirradiation DNA reconfiguration in single *recO* mutant is associated with RecBCD-mediated DNA repair. The absence of anucleate cells in *recB recO ruvABC* mutant (Table 3) indicates that inactivation of *recO* was necessary to abolish the residual chromosome segregation defect of the *recB ruvABC* mutant. The latter finding suggests that a minor part of unresolved recombination intermediates that affect nucleoid morphology in γ-irradiated *ruvABC* mutant comes from recombination processes occurring via the RecFOR pathway.

### 3.3. Effects of recB, recO, ruvABC and recG Mutations on Cell Survival and Morphology after Exposure to UV-Irradiation

**Figure 10:**
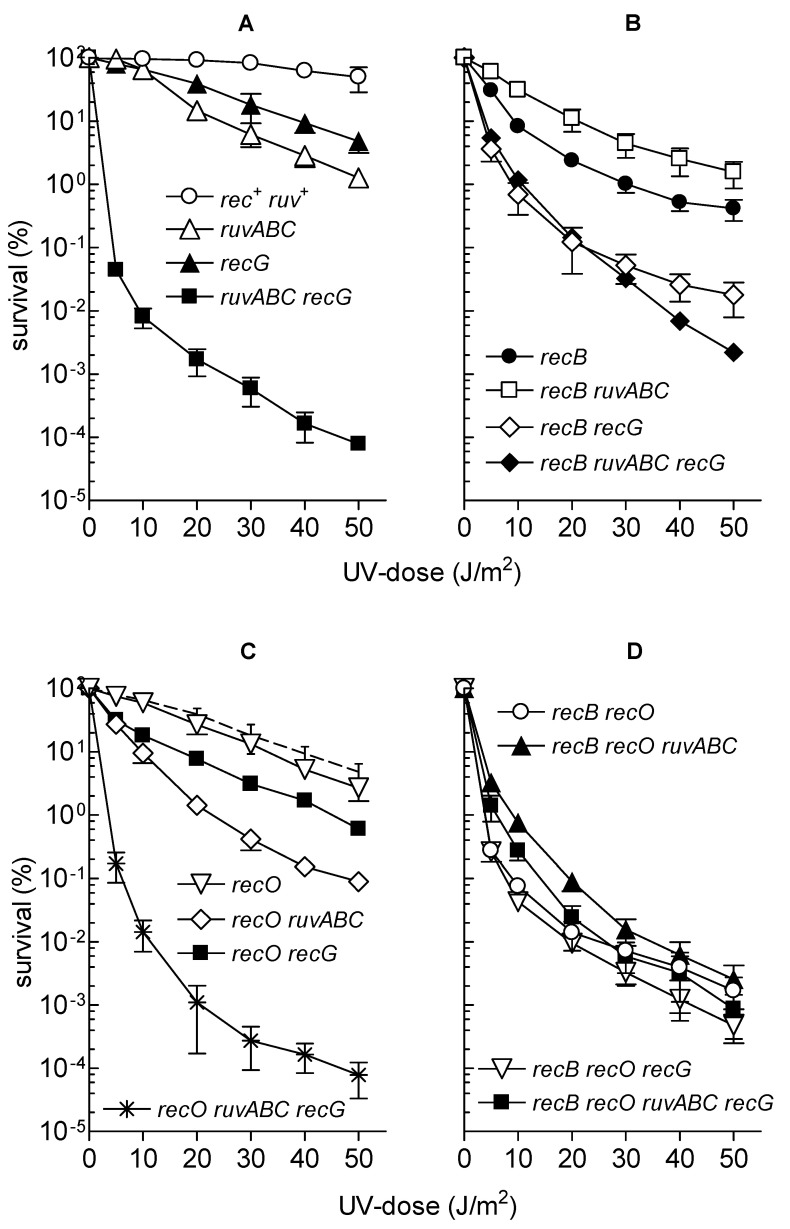
Survival of different *E. coli* mutants after UV-irradiation. The data for each strain are averages of results from at least three independent experiments, with error bars representing standard deviations. The strains used are listed in the legend to Figure 5. Dashed line on panel (**C**) represents survival of the *recG* mutant. Statistical analysis is provided in Tables S9 and S10.

UV light is a DNA-damaging agent that induces SSGs and DSBs, the DNA lesions that both require recombination to be mended [1,3,18]. The survival curves presented in Figure 9 show that single *recB* and *recO* mutations moderately reduced cell survival after exposure to UV light, whereas the combined *recB recO* mutations conferred an extreme UV sensitivity. These results are in accord with previously published data with *recB* and *recF* mutations [39]. Reminiscent of the γ-radiation survival curves, in Figure 5, the single *ruvABC* and *recG* mutants showed moderate UV sensitivity while double *ruvABC recG* mutant showed a dramatic drop of survival (Figure 10A) (see also [40]). 

The *recB ruvABC* mutant showed similar UV sensitivity as the single *recB* and *ruvABC* mutants (Figure 10B, Table S9), suggesting that RecBCD and RuvABC complexes work in the same repair pathway. In contrast, a significant synergistic effect was observed with the double *recB recG* mutant, which is in accord with previous results [41]. Furthermore, the *recB ruvABC recG* mutant was only slightly more sensitive to UV than the *recB recG* mutant (Figure 10B). Again, the *recB ruvABC recG* mutant survived significantly better than *ruvABC recG* mutant (compare Figure 10A,B), thus repeating the survival pattern observed in experiments with I-*Sce*I induction and γ-irradiation (see above). Thus, inactivation of the RecBCD enzyme precluded toxic accumulation of recombination intermediates in UV-irradiated *ruvABC recG* cells.

Combining the *recO* mutation with the *recG* mutation indicated a possible synergy in reducing cell survival (Figure 10C). The synergistic effect was pronounced with double *recO ruvABC* mutant suggesting that RuvABC largely acts outside the RecFOR pathway (i.e., on the RecBCD pathway, see above). The triple *recO ruvABC recG* mutant was equally sensitive as *ruvABC recG* mutant (Figure 10A,C). Finally, combining the *recB recO* pair of mutations with *ruvABC*, *recG*, or both, resulted in similar UV sensitivity, i.e., all the multiple mutants behaved essentially as the parental *recB recO* strain (Figure 10D).

**Figure 11:**
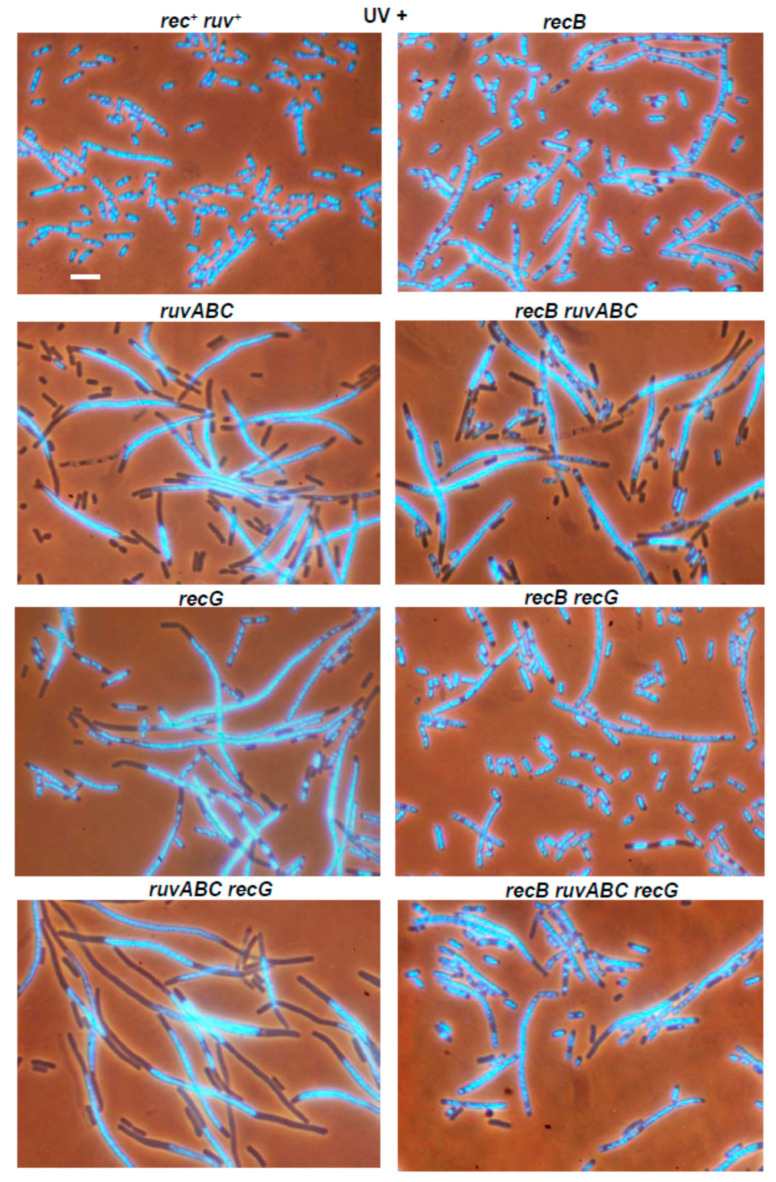

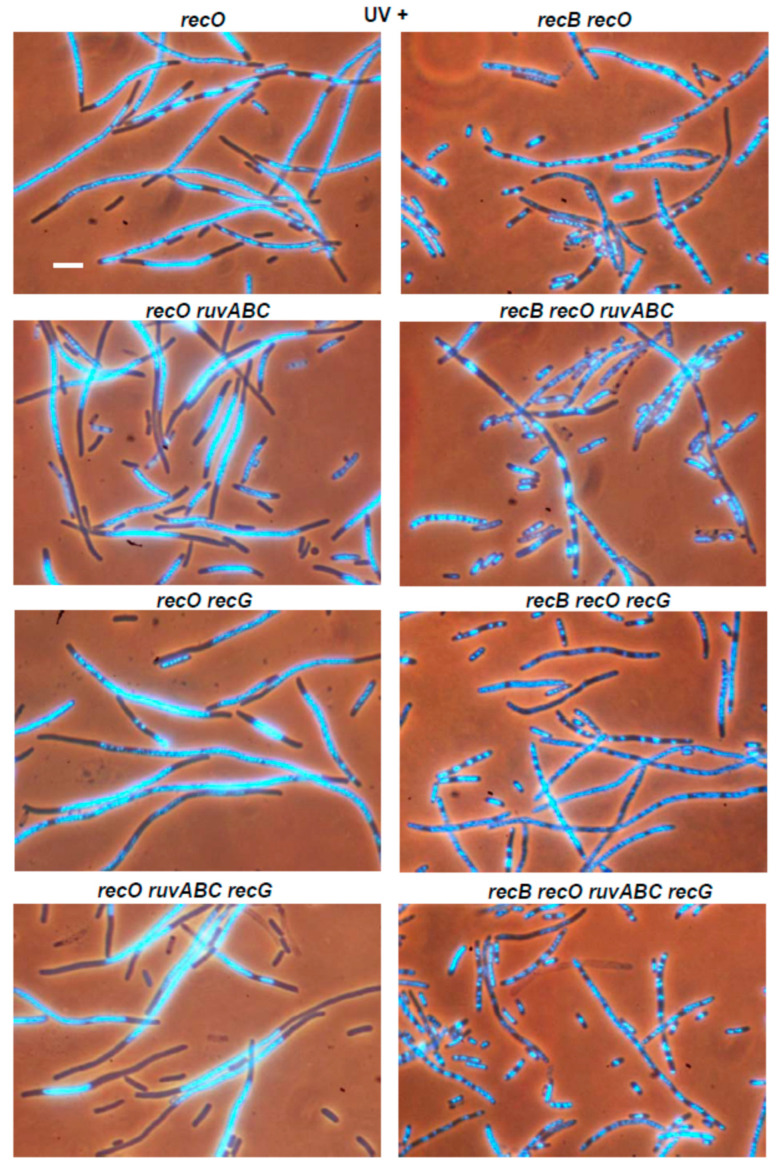
The effects of UV-irradiation on chromosome segregation and cell division in different recombination-deficient mutants of *E. coli*. Exponential cells were irradiated with 5 J/m^2^ of UV light and then further cultured for two hours prior to microscopic analysis. Unirradiated (control) cells are already shown in Figure 6, Figure 7, Figure 8 and Figure 9. Bar, 5 μm.

The applied UV dose of 5 J/m^2^ had mild effect on cellular morphology of the wild-type strain (Figure 11). As in I-*Sce*I- and γ-experiments, more dramatic effect was observed with *ruvABC* mutant, which showed pronounced chromosome segregation defect accompanied with an extreme production of anucleate cells (Figure 11, Table 3). The *recG* mutant displayed similar phenotype, although with lower production of anucleate cells. Again, the overall morphological defects were most pronounced in the double *ruvABC recG* mutant, which formed long filaments with highly condensed large nucleoids and numerous anucleate cells (Figure 11, Table 3). The above results are consistent with the results of the previous studies [28,29]. The *recB* mutation itself did not affect strongly the morphology of UV-irradiated wild-type cells, however, it almost completely abolished chromosome segregation defects in *recG* mutants (Figure 11). In accord with a previous study [29], the *recB* mutation had quite moderate effect on the morphology of UV-irradiated *ruvABC* mutants (Figure 11). The effect of the *recB* mutation was manifested mostly in an approximately 40% reduction in the number of anucleate cells in the *ruvABC* population, with an almost equal increase in the number of normal-sized cells containing DNA (Table 3). This change suggests that the *recB* mutation leads to a slight improvement in chromosome segregation in UV-irradiated *ruvABC* mutants. 

Nevertheless, the overall cytological picture of UV-irradiated *ruvABC* and *recB ruvABC* mutants is basically very similar in that the production of anucleate and filamentous cells is very high (Figure 11, Table 3). This result, together with similar UV-survival curves for *recB*, *ruvABC*, and *recB ruvABC* strains (see Figure 10A,B) is consistent with a model in which RuvABC resolvase acts prior to RecBCD in processing stalled and regressed replication forks [20,21]. 

The *recB* mutation significantly alleviated chromosome segregation defect as well as other cytological defects of *ruvABC recG* double mutant (Figure 11, Table 3). This was in accord with beneficial effect of the *recB* mutation on UV-survival of *ruvABC recG* cells. Namely, at a UV dose of 5 J/m^2^ used for microscopic experiments, the survival of *recB ruvABC recG* mutant was approximately two orders of magnitude higher than in *ruvABC recG* mutant (Figure 10A,B).

Somewhat unexpectedly, the UV-irradiated *recB ruvABC recG* mutant had less disturbed morphology than either *ruvABC* or *recB ruvABC* mutants (Figure 11). This was most obvious in significantly reduced production of anucleate cells in the triple mutant (Table 3). Additionally, the *recB ruvABC recG* mutant showed less pronounced nucleoid aggregation and cell filamentation (Figure 11, Table 3). These observations indicate that combination of *recB* and *recG* mutations partially suppresses cytological defects of *ruvABC* mutants. 

After UV-irradiation, the *recO* mutant showed pronounced morphological defects displayed as defective nucleoid partition, anucleate cell production and strong filamentation (Figure 11, Table 3). The UV-irradiated *recO ruvABC* mutant showed stronger nucleoid condensation than the single *recO* mutant. As a result, the *recO ruvABC* mutant formed filamentous cells with longer DNA-free zones next to cell poles (Figure 11). In fact, the phenotype of the *recO ruvABC* mutant was similar to that of the *ruvABC* mutant, except for a reduced number of anucleate cells in the former strain (Table 3). The reason for this difference could be in longer cell division delay in the double mutant as inferred from filamentation that was much stronger than that in the *ruvABC* mutant (Table 3). Indeed, when microscopic analysis was repeated an hour later (i.e., after 3 h of postirradiation incubation), the *recO ruvABC* mutant showed twice the production of anucleate cells and three-fold reduction in number of filaments (data not shown). In other words, the cytology of the *recO ruvABC* mutant three hours postirradiation resembles that of the *ruvABC* mutant two hours postirradiation. Cell morphology of the UV-irradiated *recO recG* double mutant was quite similar to that of the single *recO* mutant, except for a slightly increased number of filamentous cells in the double mutant (Figure 11, Table 3). Furthermore, the morphologies of the *recO ruvABC recG* and *ruvABC recG* mutants were also similar, except for the slightly reduced number of anucleate cells after the addition of the *recO* mutation. 

Finally, we investigated the morphology of the UV-irradiated *recB recO* mutant and its *ruvABC*, *recG* and *ruvABC recG* derivatives. In brief, the *recB recO* mutant and its derivatives had the same phenotype; none of these strains showed nucleoid aggregation or produced anucleate cells (Figure 11, Table 3). Hence, the *recB* mutation suppressed the aforementioned cytological defects of UV-irradiated *recO* mutants, while the *recB recO* mutation combination was necessary to achieve the same suppressive effect in *ruvABC* and *ruvABC recG* mutants. 

## 4. Discussion

In this paper, we studied cytological changes in *E. coli* cells exposed to three different DNA-damaging treatments. We were primarily focused on changes in chromosome morphology and segregation caused by DNA damage, as well as on the accompanying disorders in cell division. Cell morphology was monitored in wild-type strain and in recombination mutants carrying *recB*, *recO*, *ruvABC*, and *recG* mutations, either alone or in various combinations. The four mutations were selected assuming that two of them (*recB* and *recO*) block the initiation stage of the two major recombination pathways, while the remaining two mutations (*ruvABC* and *recG*) inactivate major recombination intermediate resolution functions. The results of microscopic analyses were correlated with survival data obtained in the accompanying DNA repair experiments. To “calibrate” our experimental system, we first performed experiments with I-*Sce*I endonuclease expression knowing that it induces exclusively plain DSBs. 

**Figure 12:**
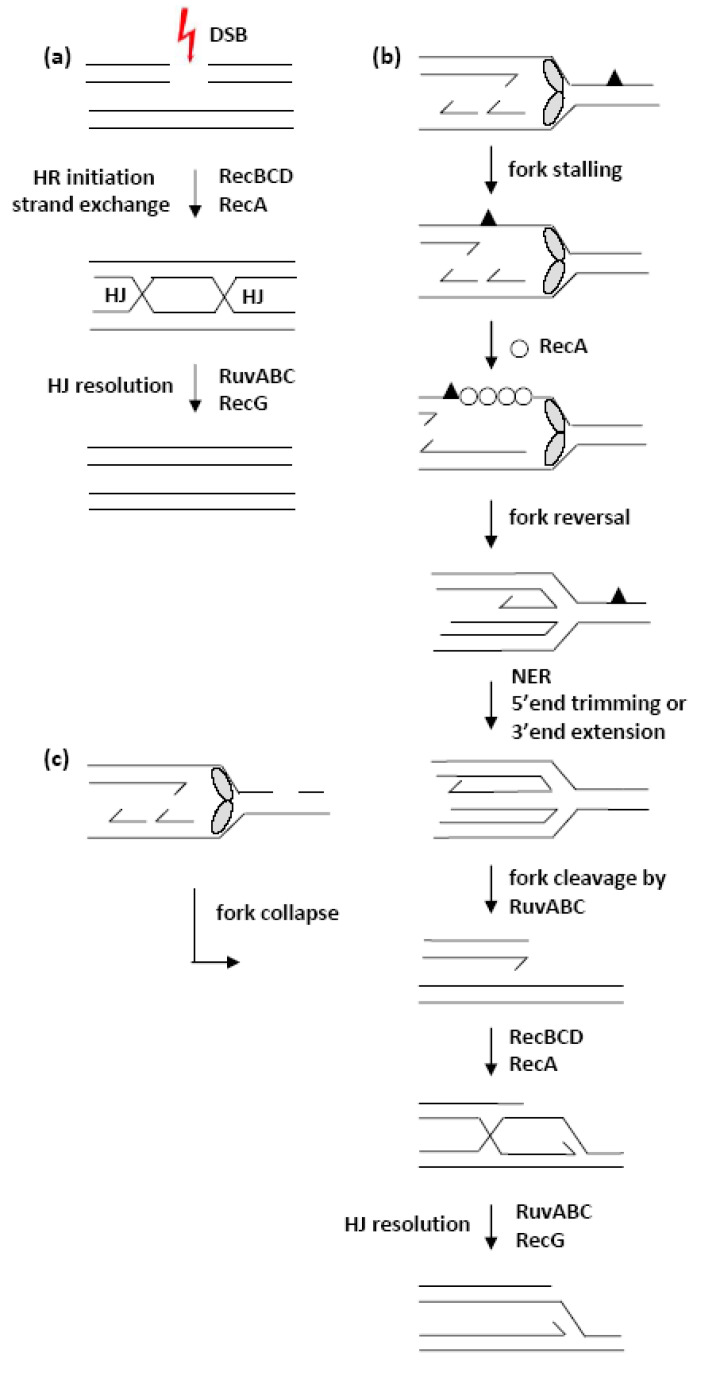
Models depicting the possible sources of DSBs and the corresponding repair pathways following the three different DNA-damaging treatments used in this work. (**a**) Classical DSB induced either by I-*Sce*I endonuclease or γ-irradiation has two dsDNA ends that are repaired by the RecBCD pathway of homologous recombination (HR). (**b**) In UV-irradiated cells, most DSBs are caused by the breakage of stalled replication forks. The drawing shows the progression of the replication fork on a DNA template damaged by UV radiation (grey ovals represent a replisome). An SSG occurs when the replication fork stops at a pyrimidine dimer (black triangle) on the leading strand template. The gap is then coated with RecA protein (white circles) in a process facilitated by the RecFOR complex [42] or an unknown factor (probably a replisome component) [21]. Homologous pairing catalysed by RecA leads to fork reversal, with concomitant replisome disassembly. Reversal of the replication fork allows NER enzymes to access the pyrimidine dimer and remove it. HJ formed by replication fork reversal is cleaved by the RuvABC resolvase thus creating a DSB. The RecBCD enzyme initiates the repair of DSB resulting in the restoration of the replication fork. (**c**) Minor sources of DSBs after UV and γ-irradiation. The replication fork runs into a single-stranded DNA interruption on the DNA template and collapses. In UV-irradiated cells, transient ssDNA breaks may arise due to ongoing NER. Alternatively, replication fork collapse can occur at unrepaired SSGs. In γ-irradiated cells, some DSBs may arise when replication fork encounters a site of ongoing base excision repair or an unsealed ssDNA break. Some ssDNA breaks could be widened by ssExos into SSGs that could also cause collapse of the replication forks [49].

Induction of DSBs by I-*Sce*I caused a chromosome partition defect and production of anucleate cells in wild-type, *ruvABC*, *recG*, and *ruvABC recG* strains, although with different intensities. While these cytological disorders were only mildly expressed in the wild-type strain, they were manifested more drastically in mutants defective for resolution of recombination intermediates (Figure 1). Inactivation of *recB* gene completely suppressed all cytological disorders in strains mentioned above (Figure 2). Additionally, the *recB* mutation was shown to be epistatic to *ruvABC*, *recG*, and *ruvABC recG* mutations during survival measurements after I-*Sce*I induction (Table 1). In contrast, introduction of the *recO* mutation proved to be neutral in DNA repair experiments, and had very little effect in the microscopic experiments (Figure 3, Table 1). Taken together, these findings clearly indicate that attempted DSB repair via the RecBCD pathway influences DNA distribution within the cell as well as its transmission upon cell division. We assume that the DNA strand exchange between sister chromosomes causes a temporary delay in chromosome segregation and disruption of cell division. In the absence of postsynaptic RuvABC and/or RecG functions, the RecBCD-mediated DSB repair leads to accumulation of recombination intermediates (Figure 12a) that more persistently interfere with chromosome partition and cell division.

The results of our experiments with γ-irradiation largely mimicked those from the I-*Sce*I induction experiments. First, γ-irradiation caused similar cytological defects as I-*Sce*I induction in wild-type, *ruvABC*, *recG*, and *ruvABC recG* strains (Figure 6, Table 3). Second, the *recB* mutation has eliminated the most of nucleoid segregation defects and anucleate cell production in the above strains (Figure 7). Third, the *recB* mutant and its *ruvABC*, *recG*, and *ruvABC recG* derivatives had generally similar γ-survival curves (Figure 5). All these findings are in accord with the assumptions that (i) DSBs are the most lethal DNA lesions caused by γ-irradiation [16], (ii) the RecBCD pathway is the main route for DSB repair in *E. coli* [1,11] (Figure 12a), and (iii) the cytological changes in γ-irradiated *E. coli* are mostly associated with RecBCD-mediated DSB repair [30] (this paper). However, our results also reveal that the RecFOR pathway affects the morphology and distribution of nucleoids in γ-irradiated cells, although to a much lesser extent than the RecBCD pathway. The RecFOR pathway was responsible for a minor part of nucleoid partition defects in γ-irradiated *ruvABC* mutant. Interestingly, these defects were also found to be RecG-dependent, suggesting that the RecG helicase acts prior to the RuvABC complex in the RecFOR pathway. These findings also imply that a small proportion of γ-induced cytological changes are associated with the repair of DNA lesions other than DSBs. Since γ-radiation causes various types of oxidative base damage [16], it is possible that some of these lesions lead to the formation of SSGs.

UV-irradiation produced the most complex cytogenetic pattern in our study. This is not surprising given that UV-irradiation causes SSGs and DSBs whose repair requires the activity of both major recombination pathways [1,18]. Recombination, or at least some of its functions, are required to rescue replication forks stalled at UV-induced pyrimidine dimers [20,21,42]. Cytological defects caused by UV-irradiation in wild-type, *ruvABC*, *recG*, and *ruvABC recG* strains were quite similar to those caused by two other DNA-damaging treatments used in this study. However, important differences were observed when *recB* or *recO* mutations were introduced in some of the above backgrounds. In particular, neither the *recB* nor the *recO* mutation alone suppressed the cytological defects of the UV-irradiated *ruvABC* mutant (Figure 11, Table 3). Suppression of these defects was achieved only by simultaneous inactivation of the *recB* and *recO* genes. Seemingly, the latter finding is consistent with the assumption that the RecBCD and RecFOR proteins operate on separate repair pathways, both of which require the RuvABC resolvase. However, given the complexity of disorders in DNA metabolism caused by UV radiation and the complexity of cellular response to these disorders, we need to consider other possible models that could explain our results. 

Similar cytology of UV-irradiated *ruvABC* and *recB ruvABC* mutants (Figure 11), as well as the similarity of their survival curves (Figure 10), is consistent with a model predicting that RuvABC resolvase acts prior to RecBCD in processing stalled and reversed replication forks [20,21] (Figure 12b). Namely, it has been suggested that part of the replication forks stalled at pyrimidine dimers move backward (driven by RecA-mediated re-annealing of template strands) and thus create Holliday junctions (HJs). By cleaving these HJs, the RuvABC resolvase produces DSBs that are repaired by the RecBCD enzyme [21] (Figure 12b). Based on this model, one could hypothesise that the cytological defects of the UV-irradiated *ruvABC* mutant result largely from unresolved HJs created by replication fork reversal. Furthermore, our results show that the *recO* mutation reduces the chromosome segregation defects present in UV-irradiated *recB ruvABC* mutant (Figure 11). As part of the above model, this could mean that the RecFOR complex participates in the reversal of the stalled replication fork and its conversion into a HJ. However, the finding that the *recO* mutation alone has only a modest effect on the cytological phenotype of the *ruvABC* mutant cannot simply fit into the above scenario. This finding is in better agreement with the results of a previous study suggesting that UV-induced reversal and breakage of replication forks is largely independent of the RecFOR complex [21]. In addition, pronounced cytological defects of *recO ruvABC* mutants are ameliorated by the *recB* mutation, suggesting that at least in the *recO* background, the RecBCD enzyme acts before the RuvABC complex.

Interestingly, the UV-irradiated *recO* mutant itself shows a significant chromosome partition defect and anucleate cell formation, both of which are efficiently suppressed by the *recB* mutation (Figure 11, Table 3). This puzzling observation could be explained by the assumption that inactivation of the RecFOR pathway leads to a redirection of DNA repair to the RecBCD pathway, presumably by conversion of non-repaired SSGs to DSBs [17,18,24] (Figure 12c). Increased RecBCD-mediated DNA repair activity could be in turn manifested on cytological level through enhanced nucleoid partition arrest and increased production of anucleate cells. Conceivably, these cytological defects would be further exacerbated by inactivation of the RuvABC resolvase. 

The idea that inactivation of the RecFOR pathway enhances formation of DSBs after UV-irradiation originates from early studies in which chromosome fragmentation was measured by sucrose gradient sedimentation in NER-deficient *uvrB* mutants and their *recF*, *recB* and *recF recB* derivatives [24,25]. Due to the extreme UV sensitivity of the strains used, chromosome fragmentation was measured at very low UV doses (up to 1 J/m^2^) [24,25]. More recently, the measurement of chromosome fragmentation in UV-irradiated *recB* cells by pulsed-field gel electrophoresis has shown that inactivation of the RecFOR pathway increases the formation of DSBs at UV doses up to 4 J/m^2^ [21]. However, at higher UV doses, the *recF recB* mutant showed a gradual decrease in chromosome fragmentation compared to its *recF^+^* counterpart [21]. The direct application of the latter findings to our work is not simple due to differences in genetic backgrounds and growth conditions of the strains used, and possibly also due to differences in dosimetry. In particular, the much stronger UV sensitivity of the conditional *recBC* (Ts) mutant used in the previous study [21] compared to the *recB* null mutant used in our work (Figure 10) raises the possibility that the latter strain accumulated less DNA lesions at nominally the same UV doses. Therefore, it is possible that, at UV dose used in our cytological experiments (i.e., at 5 J/m^2^ according to our dosimetry), the *recO* mutation enables the conversion of unrepaired SSGs to DSBs and thus enhances RecBCD-dependent DNA repair activity. 

It has been proposed that the RecFOR complex, together with the RecA protein, participates in the protection and reactivation of stalled replication forks in UV-irradiated *E. coli* cells [42,43]. This process involves a reversal of stalled forks, which enables NER enzymes access to blocking lesion. According to this model, the reversed forks remain almost intact, undergoing only limited exonucleolytic processing before replisome re-loading and replication restart [42,43]. However, as mentioned above, some experimental data strongly suggest that part of the regressed replication forks are broken by the RuvABC resolvase [20,21,40]. It has also been suggested that this latter process is largely RecFOR-independent at low UV doses [21]. Currently, it is not known which of the two mechanisms is dominant in UV-irradiated wild-type cells. It is possible that the choice of the mechanism used for rescuing stalled replication forks depends on the dose of UV radiation, i.e., the density of UV lesions on the DNA template [21]. 

The RecG protein is involved in the processing of recombination intermediates, as well as in the resetting and reactivation of stalled replication forks [44]. UV-survival curves obtained in our work suggest that RecG acts mostly in the RecFOR pathway (Figure 10). This may indicate an important role of RecG in the repair of persistent SSGs that occur when replication forks skip pyrimidine dimers on the lagging strand template. Conceivably, RecG could be required to process Holliday junctions created during RecFOR-mediated SSG repair. However, chromosome segregation defects as well as the production of anucleate cells in UV-irradiated recG single mutants appear to be entirely related to recombination in the RecBCD pathway (Figure 11). This suggests that part of the DNA repair defect of UV-irradiated recG mutants cannot be directly detected at the cytological level. Furthermore, our results show that the *recG* mutation aggravates chromosome segregation defects of UV-irradiated *ruvABC* mutants, but modestly alleviates these defects in *recB ruvABC* mutants (Figure 11, Table 3). Given that *ruvABC* and *recB ruvABC* mutants have similar UV-survival and cytology, the above findings suggest that RecG may act both prior and after the RecBCD enzyme in UV-irradiated cells. It was shown previously that inactivation of RecG slightly increases chromosome fragmentation in UV-irradiated *recB* mutants, suggesting that RecG activity to some extent counteracts replication fork reversal and/or breakage [21]. Our results suggest that in the absence of RecG, a part of UV repair (and to lesser extent also γ-repair) is re-directed toward RecBCD pathway without the mediating role of the RuvABC resolvase. We can speculate that the RecG protein is involved in the RecFOR-dependent stabilisation of stalled replication forks, and that in the absence of RecG, some of the stalled forks disintegrate independently of the RuvABC resolvase. However, our results do not rule out the involvement of RecG protein in the repair of persistent SSGs. In addition to its presumed role in processing of recombination intermediates, the RecG protein could also be involved in stabilisation of initial homologous joints during SSG repair. If so, part of the strand exchange events during SSG repair in *recG* mutants would be interrupted at an early stage, leading to SSG persistence and a higher risk of its conversion to DSBs.

The next question is how the lack of RuvABC complex affects the repair of SSGs and consequently, chromosome morphology. UV-survival curves do not show any synergistic effect of *ruvABC* and *recB* mutations (Figure 10A,B), suggesting that the RuvABC complex does not play an important role in the repair of persistent SSGs. Perhaps, the function of RuvABC at SSGs may be efficiently replaced by RecG. Nevertheless, given our finding that the *ruvABC* mutation can strongly affect cell morphology without having an equally strong effect on cell survival, it is possible that part of the morphological disorders in UV-irradiated *ruvABC* mutants is associated with defective repair of persistent SSGs. Thus, cytological disorders in UV-irradiated *ruvABC* mutants are likely the cumulative result of defects in the processing of reversed replication forks as well as defects in the repair of DSBs and persistent SSGs. In any case, the results of this work once again indicate the complexity of the effects of UV radiation on the structure and function of DNA, as well as the intertwining of recombination pathways involved in UV repair. 

All treatments that damage the chromosome and/or interfere with its replication or segregation simultaneously cause a halt in cell division (reviewed in [45]). This phenomenon is partially related to the action of the cell division inhibitor protein SulA, which is synthesised as part of the SOS response. Another inhibitory mechanism is based on the nucleoid occlusion system. This mechanism generally prevents septum formation in the cell zone occupied by chromosomal DNA, thus avoiding septation over the nucleoid (the so-called guillotine effect). In the case of defective chromosome segregation, the large nucleoid associated with the cell division inhibitor SlmA remains at midcell and interferes with septation at its normal position, leading to cell filamentation [45]. It is generally accepted that division inhibition mechanisms serve to coordinate chromosome replication and segregation with cell division when DNA metabolism is disturbed. However, none of the inhibitory mechanisms can completely prevent aberrant divisions resulting in anucleate cells or cells with “guillotined” nucleoids [45,46]. In particular, the previous and present data clearly show that the initiation of cell division often precedes chromosome segregation in cells recovering from DNA damage, thus creating a high proportion of anucleate cells in the population ([28,29,46] and this work). 

Induction of the SOS system, including the synthesis of the SulA protein, depends on the loading of the RecA protein to single-stranded DNA that is generated after DNA damage (reviewed in [47]). The loading of RecA protein to ssDNA is mediated by the RecBCD enzyme in the case of DSBs or the RecFOR complex in the case of SSGs. Accordingly, SOS-dependent inhibition of cell division after DNA damage might be expected to depend on the availability of the appropriate RecA loading complex. Indeed, expression of the *sulA* gene in cells in which DNA has been damaged by I-*Sce*I nuclease is completely dependent on the RecBCD enzyme [36], while in cells exposed to UV radiation, maximal expression of the *sulA* gene depends dominantly on the RecFOR complex [48]. A mixed situation is present in γ-irradiated cells where both recombination pathways contribute significantly to the induction of the SOS response [49].

Our experiments with I-*Sce*I expression show that filamentation is on average stronger in strains with a functional RecBCD enzyme (Table 2), which could be a combined effect of SOS (SulA)-dependent inhibition of cell division and nucleoid occlusion. Filamentation is further enhanced in strains that show a severe defect in chromosome segregation (i.e., in *ruvABC* and/or *recG* mutants), possibly due to persistent SOS-dependent division inhibition and stronger nucleoid occlusion. After expression of I-*Sce*I, the *recB* mutants are unable to induce the SOS response [36] and thus show only slight filamentation (Table 2), which is probably the result of disturbed DNA arrangement in some cells. Namely, although the *recB* cells generally do not show a strong disorder in the segregation of chromosomes, they sometimes have DNA dispersed throughout the cell volume (Figure 2), which possibly generates the nucleoid occlusion effect. The same explanation can be applied to *recB recO* mutants that show a similar phenotype as *recB* mutants (Figure 4). 

As mentioned above, in γ-irradiated cells, the induction of the SOS response depends on both RecA loading systems [49]. Therefore, a certain degree of SOS (SulA)-dependent filamentation can be expected in *recB* or *recO* single mutants after γ-irradiation, which is in agreement with our results (Table 3 and Figure 7, Figure 8, Figure 9 and Figure 11). In UV-irradiated cells the SOS response depends on the RecFOR complex [48]. However, at moderate UV doses (up to 20 J/m^2^), *recFOR* mutations cause only a short delay in the induction of the SOS response, but not its inhibition [50,51]. This explains the UV-induced filamentation we observed in both *recB* and *recO* mutants. It should be noted that in *recO* mutants, filamentation may be additionally affected by nucleoid occlusion (Figure 11). 

However, it is interesting that postirradiation filamentation is also pronounced in double *recB recO* mutants (see Table 3 and Figure 9 and Figure 11), although the double mutants should be severely deficient for SOS induction [48,49]. Additionally, both UV-irradiated and γ-irradiated *recB recO* filaments often show partial nucleoid segregation (Figure 9 and Figure 11), which should reduce the effect of nucleoid occlusion. However, it is possible that despite some nucleoids being separated, their position in the cell is not correct, thereby interfering with septation. Furthermore, since *recB recO* mutants are poorly viable [39,52] and extremely sensitive to both types of radiation ([39], this paper), it is also possible that cell filamentation occurs as a result of a general deregulation of cellular processes associated with cell dying. Finally, the possibility that there is an additional but still unknown mechanism in *E. coli* that inhibits cell division after DNA damage cannot be ruled out.

## Figures and Tables

**Table 1:** Survival of different *E. coli* mutants after I-*Sce*I endonuclease expression. Statistical analysis is provided in Tables S2 and S3.

Strain	Relevant Genotype	Survival ^a^ (30 min)	Survival ^a^ (60 min)
TRM387	*rec^+^ ruv^+^*	0.76 ± 0.099	0.39 ± 0.012
LMM4199	Δ*ruvABC*	0.68 ± 0.009	0.34 ± 0.101
LMM4201	Δ*recG*	0.19 ± 0.04	0.059 ± 0.006
LMM4202	Δ*ruvABC* Δ*recG*	0.0054 ± 0.0029	0.00088 ± 0.00067
LMM4198	Δ*recB*	0.035 ± 0.011	0.0057 ± 0.0023
LMM4611	Δ*recB* Δ*ruvABC*	0.039 ± 0.015	0.0061 ± 0.0017
LMM4612	Δ*recB* Δ*recG*	0.036 ± 0.022	0.0071 ± 0.0035
LMM4613	Δ*recB* Δ*ruvABC* Δ*recG*	0.035 ± 0.0096	0.0042 ± 0.0011
LMM4617	Δ*recO*	0.72 ± 0.021	0.38 ± 0.045
LMM4618	Δ*recO* Δ*ruvABC*	0.76 ± 0.05	0.49 ± 0.14
LMM4619	Δ*recO* Δ*recG*	0.30 ± 0.06	0.14 ± 0.012
LMM4620	Δ*recO* Δ*ruvABC* Δ*recG*	0.0043 ± 0.0013	0.0011 ± 0.0002
LMM4621	Δ*recB* Δ*recO*	0.013 ± 0.007	0.0037 ± 0.0016
LMM4658	Δ*recB* Δ*recO* Δ*ruvABC*	0.018 ± 0.009	0.0027 ± 0.0007
LMM4659	Δ*recB* Δ*recO* Δ*recG*	0.015 ± 0.0059	0.0023 ± 0.0014
LMM4660	Δ*recB* Δ*recO* Δ*ruvABC recG*	0.014 ± 0.0025	0.0047 ± 0.0018

^a^ Cell survival as measured after 30- and 60-min growth periods in 0.2% arabinose compared with identical growth periods in 0.2% glucose. The values are averages ± standard deviations of results of three independent experiments. All strains carry the arabinose-inducible *attB*::P_BAD_ I-*Sce*I expression cassette.

**Table 2:** Production of anucleate cells and filaments in exponential cultures of different *E. coli* strains before and after I-*Sce*I endonuclease expression. Statistical analysis is provided in Table S4.

Strain	Relevant Genotype	Anucleate Cells (%) ^a,b^		Filaments (%) ^a,c^	
		I-*Sce*I (-) ^d^	I-*Sce*I (+) ^d^	I-*Sce*I (-) ^d^	I-*Sce*I (+) ^d^
TRM387	*rec^+^ ruv^+^*	0	8.9	0.3	15.8
LMM4199	Δ*ruvABC*	1.0	54.5	9.3	29.5
LMM4201	Δ*recG*	0.1	28.8	1.7	33.1
LMM4202	Δ*ruvABC* Δ*recG*	3.3	53.2	7.9	36.3
LMM4198	Δ*recB*	0	0	1.3	3.0
LMM4611	Δ*recB* Δ*ruvABC*	0	0.3	6.1	8.1
LMM4612	Δ*recB* Δ*recG*	0	0	2.2	3.7
LMM4613	Δ*recB* Δ*ruvABC* Δ*recG*	0	0	2.5	6.1
LMM4617	Δ*recO*	0	7.8	2.3	15.3
LMM4618	Δ*recO* Δ*ruvABC*	1.2	50.3	8.8	23.8
LMM4619	Δ*recO* Δ*recG*	0	24.3	3.4	24.7
LMM4620	Δ*recO* Δ*ruvABC* Δ*recG*	1.8	37.5	7.9	38.7
LMM4621	Δ*recB* Δ*recO*	0.1	0.1	2.9	9.1
LMM4658	Δ*recB* Δ*recO* Δ*ruvABC*	0.1	0.3	5.0	10.4
LMM4659	Δ*recB* Δ*recO* Δ*recG*	0	0.1	3.3	11.8
LMM4660	Δ*recB* Δ*recO* Δ*ruvABC* Δ*recG*	0.1	0.2	4.7	11.5

^a^ The frequencies of the particular cell types in the total cell population were calculated from micrographs. For each strain, at least 1000 cells were counted. ^b^ Only cells showing no trace of DAPI fluorescence were considered to be anucleate. ^c^ Cells longer than 10 μm were considered filaments. ^d^ I-*Sce*I (-) and I-*Sce*I (+) denote cells in which I-*Sce*I expression is non-induced and induced, respectively.

**Table 3:** Formation of annucleate cells and filamentats in different *E. coli* mutants following γ- or UV-irradiation. Statistical analysis is provided in Tables S7 and S8.

Strain	Relevant Genotype	Annucleate Non-Irrad.	Cells (%) ^a,b^ γ-Irrad. ^d^	UV-Irrad. ^e^	Filaments Non-Irrad.	(%) ^a,c^ γ-Irrad. ^d^	UV-Irrad. ^e^
LMM2629	*rec^+^ ruv^+^*	0	2.5	0.7	0.2	8.8	2.4
LMM3188	*ruvABC*	0.7	58.3	76.4	4.6	18.6	18.1
LMM3196	*recG*	0.1	3.7	20.2	1.6	31.9	25.6
LMM3610	*ruvABC recG*	3.0	59.4	64.1	7.1	37.7	32.4
LMM3183	*recB*	0	0	0	0.4	7.2	8.7
LMM3625	*recB ruvABC*	0.2	11.3	43.5	3.4	19.1	17.7
LMM3682	*recB recG*	0	0.3	0.1	0.8	6.1	10.5
LMM4134	*recB ruvABC recG*	0	0.4	15.0	1.3	12.4	11.6
LMM3545	*recO*	0.1	9.3	21.5	0.8	13.8	53.5
LMM3600	*recO ruvABC*	1.2	61.4	35.3	2.5	21.2	52.0
LMM3601	*recO recG*	0.2	14.7	21.9	3.5	25.0	60.7
LMM3604	*recO ruvABC recG*	4.6	51.9	55.9	5.2	33.6	34.3
LMM3599	*recB recO*	0.1	0.1	0.3	1.6	21.6	21.7
LMM3602	*recB recO ruvABC*	0	0	1.2	2.1	20.3	21.0
LMM3603	*recB recO recG*	0	0	0.4	2.2	21.2	21.8
LMM3605	*recB recO ruvABC recG*	0.1	0.1	0.2	1.7	19.1	16.6

^a^ The frequencies of the particular cell types in the total cell population were calculated from micrographs. For each strain, at least 1000 cells were counted. ^b^ Only cells showing no trace of DAPI fluorescence were considered to be anucleate. ^c^ Cells longer than 10 μm were considered filaments. ^d^ Cells were irradiated with γ-dose of 100 Gy. ^e^ Cells were irradiated with UV dose of 5 J/m^2^.

## Data Availability

All data are provided in the article and in the Supplementary Materials.

## Supplementary Materials

The following supporting information can be downloaded at: https://www.mdpi.com/article/10.3390/microorganisms11030701/s1, Table S1: *E. coli* strains used in this work. Figure S1. Growth of different TRM387 derivatives. Figure S2. Effects of γ- and UV-irradiation on growth rates of different recombination mutants of *E. coli*. Figure S3. Formation of anucleate cells in *ruvABC* mutants after introduction of double-strand DNA breaks by I-SceI endonuclease. Table S2. *E. coli* strains divided into groups (Tukey HSD test, alpha = 0.05) based on the data on survival at 60 min after I-*Sce*I expression (Table 1). Table S3. Statistical analysis of differences in survival at 60 min after I-*Sce*I expression (Table 1) presented as pairwise *p*-values (*p* adj) from the Tukey HSD test, which controls for multiple testing. Table S4. Statistical analysis of differences in the occurrence of filamentous, anucleate and regular cells in different strains after 60 min of I-*Sce*I expression (Table 2). Table S5. *E. coli* strains divided into groups (Tukey HSD test, alpha = 0.05) based on the data analysed on γ-survival at 200 Gy (see also Figure 4). Table S6. Statistical analysis of differences in survival after 200 Gy of γ-irradiation (see also Figure 4) presented as pairwise *p*-values (*p* adj) from the Tukey HSD test, which controls for multiple testing. Table S7. Statistical analysis of differences in the occurrence of filamentous, anucleate and regular cells in different strains after UV dose of 5 J/m^2^ (Table 3). Table S8. Statistical analysis of differences in the occurrence of filamentous, anucleate and regular cells in different strains after γ-dose of 100 Gy (Table 3). Table S9. *E. coli* strains divided into groups (Tukey HSD test, alpha = 0.05) based on the data on UV-survival at 50 J/m^2^ (see also Figure 9). Table S10. Statistical analysis of differences in survival after 50 J/m^2^ of UV (see also Figure 9) presented as pairwise *p*-values (*p* adj) from the Tukey HSD test, which controls for multiple testing.

## References

[1] Spies M., Kowalczykowski S.C., Higgins N.P. (2005). Homologous recombination by the RecBCD and RecF pathways. The Bacterial Chromosome.

[2] Kuzminov A. (2011). Homologous Recombination—Experimental Systems, Analysis and Significance. EcoSal Plus.

[3] Michel B., Leach D. (2013). Homologous Recombination—Enzymes and Pathways. EcoSal Plus.

[4] Mehta A., Haber J.E. (2014). Sources of DNA double-strand breaks and models of recombinational DNA repair. Cold Spring Harb. Perspect. Biol..

[5] Cox M.M. (2007). Regulation of bacterial RecA protein function. Crit. Rev. Biochem. Mol. Biol..

[6] West S.C. (1997). Processing of recombination intermediates by the RuvABC proteins. Annu. Rev. Genet..

[7] Lloyd R.G. (1991). Conjugational recombination in resolvase-deficient ruvC mutants of *Escherichia coli* K-12 depends on recG. J. Bacteriol..

[8] Meddows T.R., Savory A.P., Lloyd R.G. (2004). RecG helicase promotes DNA double-strand break repair. Mol. Microbiol..

[9] Rudolph C.J., Upton A.L., Briggs G.S., Lloyd R.G. (2010). Is RecG a general guardian of the bacterial genome?. DNA Repair..

[10] Cox M.M. (2001). Recombinational DNA repair of damaged replication forks in *Escherichia coli*: Questions. Annu. Rev. Genet..

[11] Dillingham M.S., Kowalczykowski S.C. (2008). RecBCD enzyme and the repair of double-stranded DNA breaks. Microbiol. Mol. Biol. Rev..

[12] Dixon D.A., Kowalczykowski S.C. (1993). The recombination hotspot chi is a regulatory sequence that acts by attenuating the nuclease activity of the *E. coli* RecBCD enzyme. Cell.

[13] Anderson D.G., Kowalczykowski S.C. (1997). The translocating RecBCD enzyme stimulates recombination by directing RecA protein onto ssDNA in chi-regulated manner. Cell.

[14] Arnold D.A., Kowalczykowski S.C. (2000). Facilitated loading of RecA protein is essential to recombination by RecBCD enzyme. Genes Dev..

[15] Morimatsu K., Kowalczykowski S.C. (2003). RecFOR proteins load RecA protein onto gapped DNA to accelerate DNA strand exchange: A universal step of recombinational repair. Mol. Cell.

[16] Friedberg E.C., Walker G.C., Siede W., Wood R.D., Schultz R.A., Ellenberger T. (2006). DNA Repair and Mutagenesis.

[17] Smith K.C., Wang T.-C.V. (1989). recA-dependent DNA repair processes. Bioessays.

[18] Kuzminov A. (1999). Recombinational repair of DNA damage in *Escherichia coli* and bacteriophage λ. Microbiol. Mol. Biol. Rev..

[19] Persky N.S., Lovett S.T. (2008). Mechanisms of recombination: Lessons from *E. coli*. Crit. Rev. Biochem. Mol. Biol..

[20] McGlynn P., Lloyd R.G. (2000). Modulation of RNA polymerase by (p)ppGpp reveals a RecG-dependent mechanism for replication fork progression. Cell.

[21] Khan S.R., Kuzminov A. (2012). Replication forks stalled at ultraviolet lesions are rescued via RecA and RuvABC protein-catalyzed disintegration in *Escherichia coli*. J. Biol. Chem..

[22] Bonura T., Smith K.C. (1975). Enzymatic production of deoxyribonucleic acid double-strand breaks after ultraviolet irradiation of *Escherichia coli* K-12. J. Bacteriol..

[23] Thoms B., Wackernagel W. (1998). Interaction of RecBCD enzyme with DNA at double-strand breaks produced in UV-irradiated *Escherichia coli*: Requirement for DNA end processing. J. Bacteriol..

[24] Wang T.-C.V., Smith K.C. (1983). Mechanisms for recF-dependent and recB-dependent pathways of postreplication repair in UV-irradiated *Escherichia coli* uvrB. J. Bacteriol..

[25] Wang T.-C.V., Smith K.C. (1986). Postreplicational formation and repair of DNA double-strand breaks in UV-irradiated *Escherichia coli* uvrB cells. Mutat. Res..

[26] Smith B.T., Grossman A.D., Walker G.C. (2002). Localization of UvrA and effect of DNA damage on the chromosome of *Bacillus subtilis*. J. Bacteriol..

[27] Odsbu I., Skarstad K. (2014). DNA compaction in the early part of the SOS response is dependent on RecN and RecA. Microbiology.

[28] Ishioka K., Iwasaki H., Shinagawa H. (1997). Roles of the recG gene product of *Escherichia coli* in recombination repair: Effects of the ΔrecG gene mutation on cell division and chromosome partition. Genes Genet. Syst..

[29] Ishioka K., Fukuoh A., Iwasaki H., Nakata A., Shinagawa H. (1998). Abortive recombination in *Escherichia coli* ruv mutants blocks chromosome partitioning. Genes Cells.

[30] Buljubašić M., Zahradka D., Zahradka K. (2013). RecQ helicase acts before RuvABC, RecG and XerC proteins during recombination in recBCD sbcBC mutants of *Escherichia coli*. Res. Microbiol..

[31] Levin-Zaidman S., Frenkiel-Krispin D., Shimoni E., Sabanay I., Wolf S.G., Minsky A. (2000). Ordered intracellular RecA-DNA assemblies: A potential site of in vivo RecA-mediated activities. Proc. Natl. Acad. Sci. USA.

[32] Zahradka D., Vlahović K., Petranović M., Petranović D. (1999). Chromosome segregation and cell division defects in recBC sbcBC ruvC mutants of *Escherichia coli*. J. Bacteriol..

[33] Xia J., Chen L.-T., Mei Q., Ma C.-H., Halliday J.A., Lin H.Y., Magnan D., Pribis J.P., Fitzgerald D.M., Hamilton H.M. (2016). Holliday junction trap shows how cells use recombination and a junction-guardian role of RecQ helicase. Sci. Adv..

[34] Bachmann B.J., Neidhardt F.C., Curtiss R. (1996). Derivations and genotypes of some mutant derivatives of *Escherichia coli* K-12. Escherichia coli and Salmonella: Cellular and Molecular Biology.

[35] Miller J.H. (1992). A Short Course in Bacterial Genetics.

[36] Vlašić I., Ivančić-Baće I., Imešek M., Mihaljević B., Brčić-Kostić K. (2008). RecJ nuclease is required for SOS induction after introduction of a double-strand break in a RecA loading deficient recB mutant of *Escherichia coli*. Biochimie.

[37] Vlašić I., Šimatović A., Brčić-Kostić K. (2012). The hybrid recombinational repair pathway operates in a χ activity deficient recC1004 mutant of *Escherichia coli*. Biochimie.

[38] Feliciello I., Zahradka D., Zahradka K., Ivanković S., Puc N., Đermić D. (2018). RecF, UvrD, RecX and RecN proteins suppress DNA degradation at DNA double-strand breaks in *Escherichia coli*. Biochimie.

[39] Buljubašić M., Hlevnjak A., Repar J., Đermić D., Filić V., Weber I., Zahradka K., Zahradka D. (2019). RecBCD-RecFOR-independent pathway of homologous recombination in *Escherichia coli*. DNA Repair..

[40] Bolt E.L., Lloyd R.G. (2002). Substrate specificity of RusA resolvase reveals the DNA structures targeted by RuvAB and RecG in vivo. Mol Cell.

[41] Gregg A.V., McGlynn P., Jaktaji R.P., Lloyd R.G. (2002). Direct rescue of stalled DNA replication forks via the combined action of PriA and RecG helicase activities. Mol. Cell.

[42] Courcelle J., Hanawalt P.C. (2003). RecA-dependent recovery of arrested DNA replication forks. Annu. Rev. Genet..

[43] Rudolph C.J., Upton A.L., Lloyd R.G. (2008). Maintaining replication fork integrity in UV-irradiated *Escherichia coli* cells. DNA Repair..

[44] Michel B., Sinha A.K., Leach D.R.F. (2018). Replication fork breakage and restart in *Escherichia coli*. Microbiol. Mol. Biol. Rev..

[45] Rothfield L., Taghbalout A., Shih Y.-L. (2005). Spatial control of bacterial division-site placement. Nat. Rev. Microbiol..

[46] Raghunathan S., Chimthanawala A., Krishna S., Vecchiarelli A.G., Badrinarayanan A. (2020). Asymmetric chromosome segregation and cell division in DNA damage-induced bacterial filaments. Mol. Biol. Cell.

[47] Vlašić I., Brčić-Kostić K. (2014). Rescuing a sinking ship: The role of recombination gene products in SOS induction in *Escherichia coli*. Period. Biol..

[48] Ivančić-Baće I., Vlašić I., Salaj-Šmic E., Brčić-Kostić K. (2006). Genetic evidence for the requirement of RecA loading activity in SOS induction after UV irradiation in *Escherichia coli*. J. Bacteriol..

[49] Serment-Guerrero J., Breña-Valle M., Espinosa-Aguirre J.J. (2008). In vivo role of *Escherichia coli* single-strand exonucleases in SOS induction by gamma radiation. Mutagenesis.

[50] Hegde S., Sandler S.J., Clark A.J., Madiraju M.V.V.S. (1995). recO and recR mutants delay induction of the SOS response in *Escherichia coli*. Mol. Gen. Genet..

[51] Whitby M.C., Lloyd R.G. (1995). Altered SOS induction associated with mutations in recF, recO and recR. Mol. Gen. Genet..

[52] Sinha A.K., Possoz C., Leach D.R.F. (2020). The roles of bacterial double-strand break repair proteins in chromosomal DNA replication. FEMS Microbiol. Rev..

