# Supplementary material for: Chromosome Segregation and Cell Division Defects in Escherichia coli Recombination Mutants Exposed to Different DNA-Damaging Treatments
Source: Microorganisms. 2023 Mar 9;11(3):701. doi: 10.3390/microorganisms11030701 (PMC10051365; doi:10.3390/microorganisms11030701)
Supplement: Supplementary file 1 [file microorganisms-11-00701-s001.zip › microorganisms-2273887-supplementary.pdf]

**Table S1.** *E. coli* strains used in this work.

| Strain | Relevant genotype | Source or reference |
|---|---|---|
| **Used for strain construction** | | |
| CAG18486 | *eda-51*::Tn*10* | CGSC[a] |
| JW2549-1 | Δ*recO737*::*kan* | CGSC[a] |
| LMM1672 | *recB268*::Tn*10* | Buljubašić et al., 2013 |
| LMM1673 | Δ*ruvABC*::*cam* | Buljubašić et al., 2013 |
| LMM4194 | *eda-51*::Tn*10* Δ*ruvABC*::*cam* | P1.LMM1673 × CAG18486 to $Cm^r$ $Tc^r$ $UV^s$ |
| N6052 | Δ*recG1919*::*apra* | R.G. Lloyd |
| N6197 | Δ*recB1910*::*dhfr* | R.G. Lloyd |
| **MG1655 derivatives[b] used for experiments** | | |
| LMM2629[c] | *rec*$^+$ *ruv*$^+$ | Buljubašić et al., 2013 |
| LMM3183 | *recB268*::Tn*10* | P1. LMM1672 × LMM2629 to $Tc^r$ $UV^s$ |
| LMM3188 | Δ*ruvABC*::*cam* | P1. LMM1673 × LMM2629 to $Cm^r$ $UV^s$ |
| LMM3196 | Δ*recG1919*::*apra* | P1.N6052 × LMM2629 to $Apra^r$ $UV^s$ |
| LMM3545 | Δ*recO737*::*kan* | P1. JW2549-1 × LMM2629 to $Km^r$ $UV^s$ |
| LMM3599 | Δ*recO737*::*kan* *recB268*::Tn*10* | P1.LMM1672 × LMM3545 to $Tc^r$ $UV^s$ |
| LMM3600 | Δ*recO737*::*kan* Δ*ruvABC*::*cam* | P1.LMM1673 × LMM3545 to $Cm^r$ $UV^s$ |
| LMM3601 | Δ*recO737*::*kan* Δ*recG1919*::*apra* | P1.N6052 × LMM3545 to $Apra^r$ $UV^s$ |
| LMM3602 | Δ*recO737*::*kan* Δ*ruvABC*::*cam* *recB268*::Tn*10* | P1.LMM1672 × LMM3600 to $Tc^r$ $UV^s$ |
| LMM3603 | Δ*recO737*::*kan* Δ*recG1919*::*apra* | P1.LMM1672 × LMM3601 |

| Strain | Relevant genotype | Source or reference |
|---|---|---|
| | *recB268*::Tn*10* | to Tc$^r$ UV$^s$ |
| LMM3604 | Δ*recO737*::*kan* Δ*recG1919*::*apra* Δ*ruvABC*::*cam* | P1.LMM1673 × LMM3601 to Cm$^r$ UV$^s$ |
| LMM3605 | Δ*recO737*::*kan* Δ*recG1919*::*apra* Δ*ruvABC*::*cam* *recB268*::Tn*10* | P1.LMM1672 × LMM3604 to Tc$^r$ |
| LMM3610 | Δ*ruvABC*::*cam* Δ*recG1919*::*apra* | P1.N6052 × LMM3188 to Apra$^r$ UV$^s$ |
| LMM3625 | Δ*ruvABC*::*cam* *recB268*::Tn*10* | P1.LMM1672 × LMM3188 to Tc$^r$ |
| LMM3626 | Δ*recG1919*::*apra* *recB268*::Tn*10* | P1.LMM1672 × LMM3196 to Tc$^r$ UV$^s$ |
| LMM4134 | Δ*ruvABC*::*cam* Δ*recG1919*::*apra* *recB268*::Tn*10* | P1.LMM1672 × LMM3610 to Tc$^r$ |
| TRM387 | Δ*argE*::I-*Sce*I$_{CS}$-*cat* Δ*attB*::P$_{BAD}$ I-*Sce*I | Meddows et al., 2004 |
| LMM4198 | as TRM387 plus Δ*recB1910*::*dhfr* | P1.N6197 × TRM387 to Tm$^r$ UV$^s$ |
| LMM4199 | as TRM387 plus *eda-51*::Tn*10* Δ*ruvABC*::*cam* | P1.LMM4194 × TRM387 to Tc$^r$ UV$^s$ |
| LMM4201 | as TRM387 plus Δ*recG1919*::*apra* | P1.N6052 × TRM387 to Apra$^r$ UV$^s$ |
| LMM4202 | as TRM387 plus *eda-51*::Tn*10* Δ*ruvABC*::*cam* Δ*recG1919*::*apra* | P1.N6052 × LMM4199 to Apra$^r$ UV$^s$ |
| LMM4611 | as TRM387 plus *eda-51*::Tn*10* Δ*ruvABC*::*cam* Δ*recB1910*::*dhfr* | P1.N6197 × LMM4199 to Tm$^r$ |
| LMM4612 | as TRM387 plus Δ*recG1919*::*apra* Δ*recB1910*::*dhfr* | P1.N6197 × LMM4201 to Tm$^r$ UV$^s$ |
| LMM4613 | as TRM387 plus *eda-51*::Tn*10* Δ*ruvABC*::*cam* Δ*recG1919*::*apra* Δ*recB1910*::*dhfr* | P1.N6197 × LMM4202 to Tm$^r$ |

| Strain | Relevant genotype | Source or reference |
|---|---|---|
| LMM4617 | as TRM387 plus Δ*recO737*::*kan* | P1. JW2549-1 × TRM387 to $Km^r$ $UV^s$ |
| LMM4618 | as TRM387 plus Δ*recO737*::*kan eda-51*::Tn*10* Δ*ruvABC*::*cam* | P1.LMM4194 × LMM4617 to $Tc^r$ $UV^s$ |
| LMM4619 | as TRM387 plus Δ*recO737*::*kan* Δ*recG1919*::*apra* | P1.N6052 × LMM4617 to $Apra^r$ $UV^s$ |
| LMM4620 | as TRM387 plus Δ*recO737*::*kan eda-51*::Tn*10* Δ*ruvABC*::*cam* Δ*recG1919*::*apra* | P1.N6052 × LMM4618 to $Apra^r$ $UV^s$ |
| LMM4621 | as TRM387 plus Δ*recO737*::*kan* Δ*recB1910*::*dhfr* | P1.N6197 × LMM4617 to $Tm^r$ $UV^s$ |
| LMM4658 | as TRM387 plus Δ*recO737*::*kan eda-51*::Tn*10* Δ*ruvABC*::*cam* Δ*recB1910*::*dhfr* | P1.N6197 × LMM4618 to $Tm^r$ $UV^s$ |
| LMM4659 | as TRM387 plus Δ*recO737*::*kan* Δ*recG1919*::*apra* Δ*recB1910*::*dhfr* | P1.N6197 × LMM4619 to $Tm^r$ $UV^s$ |
| LMM4660 | as TRM387 plus Δ*recO737*::*kan eda-51*::Tn*10* Δ*ruvABC*::*cam* Δ*recG1919*::*apra* Δ*recB1910*::*dhfr* | P1.N6197 × LMM4620 to $Tm^r$ |

[a] Strain obtained from the *Escherichia coli* Genetic Stock Center, Yale.

[b] MG1655 markers are $F^-$ $\lambda^-$ *ilvG rfb-50 rph-1*.

[c] As MG1655 but Δ*proA*::*frt*.

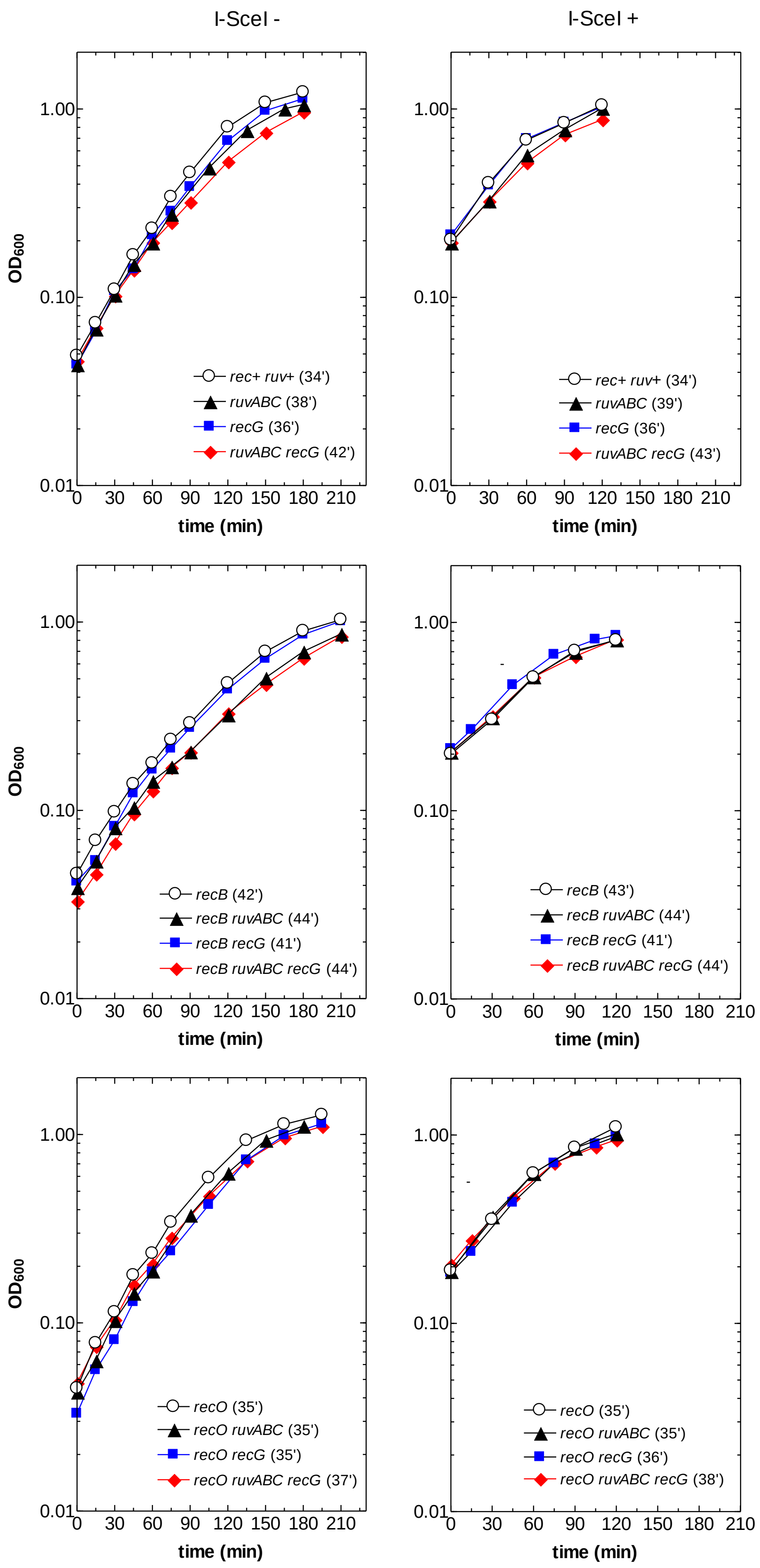

I-SceI -
I-SceI +
OD600
1.00
0.10
0.01
0 30 60 90 120 150 180 210
time (min)
rec+ ruv+ (34')
ruvABC (38')
recG (36')
ruvABC recG (42')
rec+ ruv+ (34')
ruvABC (39')
recG (36')
ruvABC recG (43')
recB (42')
recB ruvABC (44')
recB recG (41')
recB ruvABC recG (44')
recB (43')
recB ruvABC (44')
recB recG (41')
recB ruvABC recG (44')
recO (35')
recO ruvABC (35')
recO recG (35')
recO ruvABC recG (37')
recO (35')
recO ruvABC (35')
recO recG (36')
recO ruvABC recG (38')

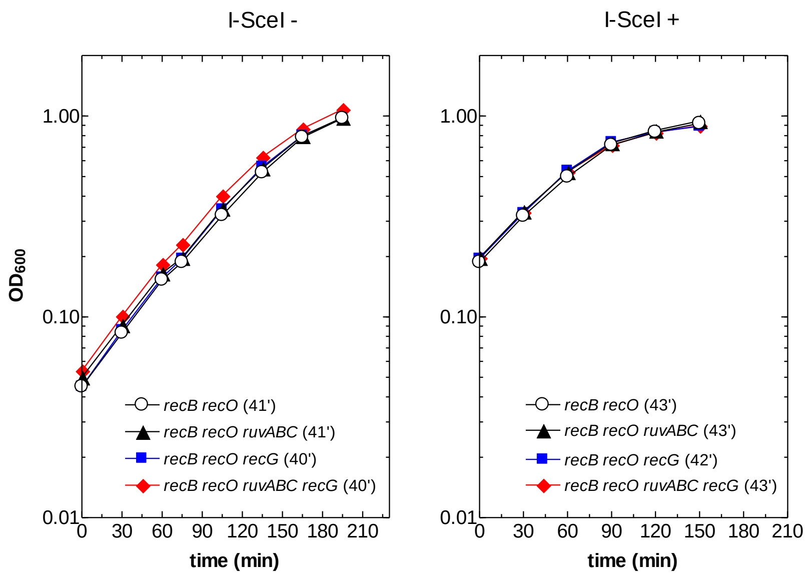


**Figure S1.** Growth of different TRM387 derivatives. Left side of the panel: normal growth in LB medium (without I-*Sce*I expression). Right side of the panel: growth in LB medium with arabinose (induced I-*Sce*I expression). To compare growth rates of I-*Sce*I-induced and -noninduced cells, mass doubling times (in minutes) were calculated from the same range of growth curves ($OD_{600}$ of 0.2 - 0.6) and are given in parentheses. Details on growth conditions are given in Materials and Methods.

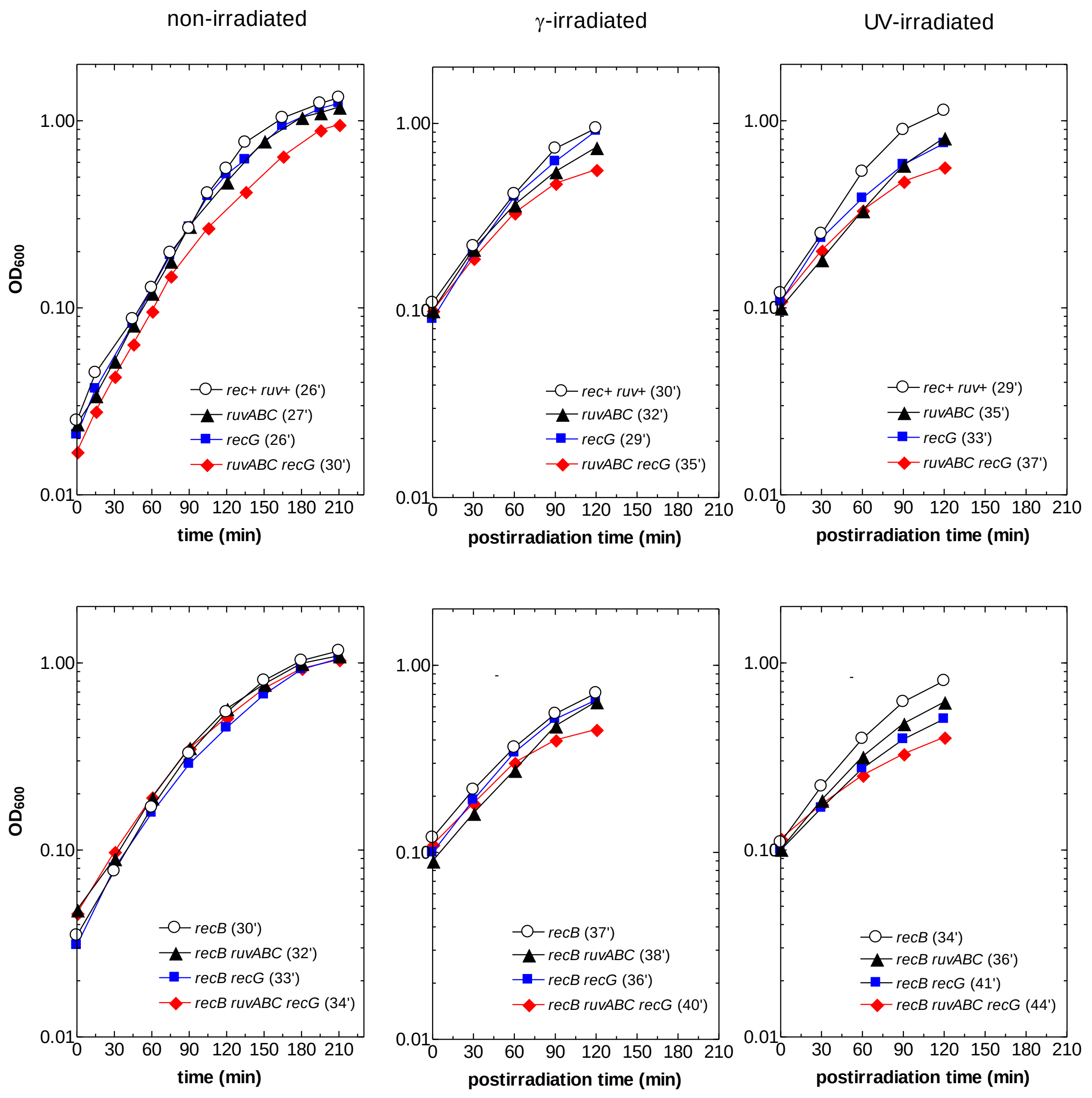


**Figure S2.** Effects of γ-irradiation (100 Gy) and UV-irradiation (5 J/m$^2$) on growth rates of different recombination mutants of *E. coli*. Mass doubling times (in minutes) were calculated from exponential parts of growth curves ($OD_{600}$ of 0.1 – 0.3) and are given in parentheses. Irradiated cell cultures were adjusted to $OD_{600}$ of ~ 0.1 and their growth was then followed for next 120 min. Details on γ- and UV-irradiation are given in Materials and Methods.

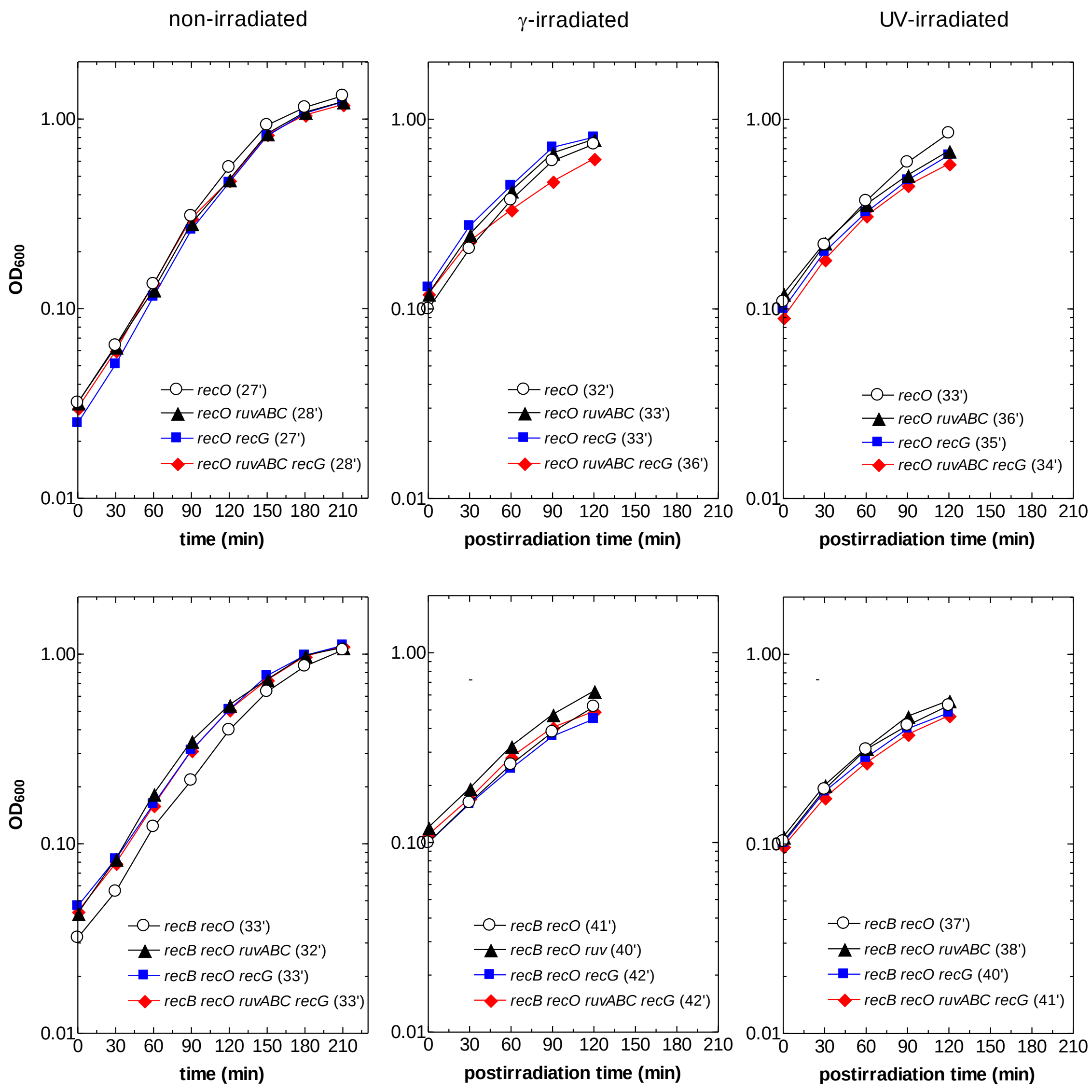


**Figure S2. Continued**

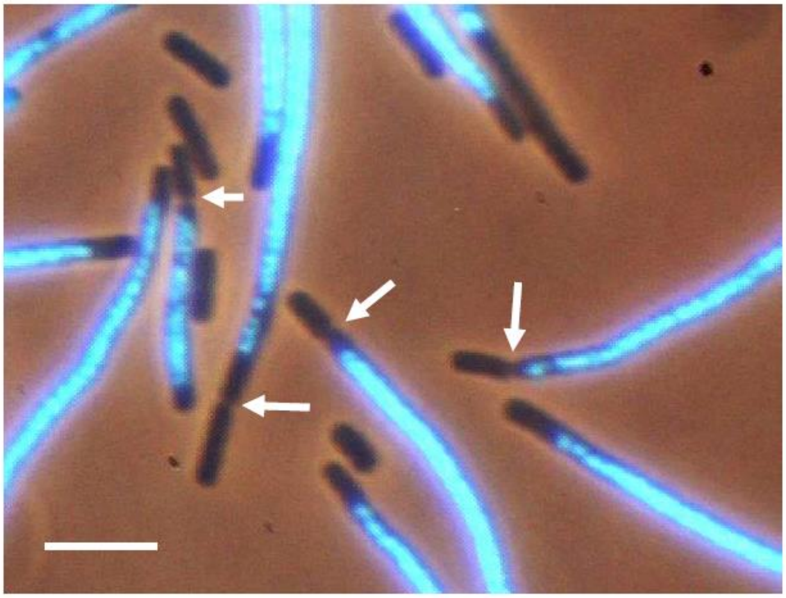

**Figure S3.** Formation of anucleate cells in *ruvABC* mutants after introduction of double-strand DNA breaks by I-SceI endonuclease. Arrows indicate septation events (cell constrictions) leading to segregation of anucleate cells. Bar, 5µm.

**Table S2.** *E. coli* strains divided into groups (Tukey HSD test, alpha=0.05) based on the data on survival at 60 min after I-*Sce*I expression (Table 1). Groups labeled with identical letters don't differ significantly in mean log survival rates. The group labels are ordered by the decreasing survival rates.

| **Strain/genotype** | **Group** |
|---|---|
| 4618_recO_ruvABC | a |
| TRM387_wt | a |
| 4617_recO | a |
| 4199_ruvABC | a |
| 4619_recO_recG | ab |
| 4201_recG | b |
| 4612_recB_recG | c |
| 4611_recB_ruvABC | c |
| 4198_recB | c |
| 4660_recB_recO_ruvABC_recG | cd |
| 4613_recB_ruvABC_recG | cd |
| 4621_recB_recO | cd |
| 4658_recB_recO_ruvABC | cde |
| 4659_recB_recO_recG | cde |
| 4620_recO_ruvABC_recG | de |
| 4202_ruvABC_recG | e |

**Table S3.** Statistical analysis of differences in survival at 60 min after I-*Sce*I expression (Table 1) presented as pairwise p-values (p adj) from the Tukey HSD test, which controls for multiple testing.

| **Comparison strain1-strain2** | **p adj** |
|---|---|
| 4199_ruvABC-4198_recB | 8.13E-09 |
| 4201_recG-4198_recB | 0.000378 |
| 4202_ruvABC_recG-4198_recB | 0.001844 |
| 4611_recB_ruvABC-4198_recB | 1 |
| 4612_recB_recG-4198_recB | 1 |
| 4613_recB_ruvABC_recG-4198_recB | 0.999999 |
| 4617_recO-4198_recB | 3.32E-09 |
| 4618_recO_ruvABC-4198_recB | 1.00E-09 |
| 4619_recO_recG-4198_recB | 1.18E-06 |
| 4620_recO_ruvABC_recG-4198_recB | 0.049389 |
| 4621_recB_recO-4198_recB | 0.999528 |
| 4658_recB_recO_ruvABC-4198_recB | 0.948694 |
| 4659_recB_recO_recG-4198_recB | 0.344266 |
| 4660_recB_recO_ruvABC_recG-4198_recB | 1 |
| TRM387_wt-4198_recB | 2.76E-09 |
| 4201_recG-4199_ruvABC | 0.026087 |

| | |
|---|---|
| 4202_ruvABC_recG-4199_ruvABC | 2.53E-13 |
| 4611_recB_ruvABC-4199_ruvABC | 1.45E-08 |
| 4612_recB_recG-4199_ruvABC | 2.32E-08 |
| 4613_recB_ruvABC_recG-4199_ruvABC | 1.81E-09 |
| 4617_recO-4199_ruvABC | 1 |
| 4618_recO_ruvABC-4199_ruvABC | 0.999931 |
| 4619_recO_recG-4199_ruvABC | 0.854895 |
| 4620_recO_ruvABC_recG-4199_ruvABC | 1.92E-12 |
| 4621_recB_recO-4199_ruvABC | 7.00E-10 |
| 4658_recB_recO_ruvABC-4199_ruvABC | 1.62E-10 |
| 4659_recB_recO_recG-4199_ruvABC | 1.37E-11 |
| 4660_recB_recO_ruvABC_recG-4199_ruvABC | 2.52E-09 |
| TRM387_wt-4199_ruvABC | 1 |
| 4202_ruvABC_recG-4201_recG | 7.09E-10 |
| 4611_recB_ruvABC-4201_recG | 0.000719 |
| 4612_recB_recG-4201_recG | 0.00121 |
| 4613_recB_ruvABC_recG-4201_recG | 6.77E-05 |
| 4617_recO-4201_recG | 0.010346 |
| 4618_recO_ruvABC-4201_recG | 0.002746 |
| 4619_recO_recG-4201_recG | 0.768763 |
| 4620_recO_ruvABC_recG-4201_recG | 1.58E-08 |
| 4621_recB_recO-4201_recG | 2.22E-05 |
| 4658_recB_recO_ruvABC-4201_recG | 3.87E-06 |
| 4659_recB_recO_recG-4201_recG | 1.90E-07 |
| 4660_recB_recO_ruvABC_recG-4201_recG | 9.94E-05 |
| TRM387_wt-4201_recG | 0.008489 |
| 4611_recB_ruvABC-4202_ruvABC_recG | 0.000976 |
| 4612_recB_recG-4202_ruvABC_recG | 0.00058 |
| 4613_recB_ruvABC_recG-4202_ruvABC_recG | 0.009574 |
| 4617_recO-4202_ruvABC_recG | 1.78E-13 |
| 4618_recO_ruvABC-4202_ruvABC_recG | 1.30E-13 |
| 4619_recO_recG-4202_ruvABC_recG | 6.91E-12 |
| 4620_recO_ruvABC_recG-4202_ruvABC_recG | 0.994556 |
| 4621_recB_recO-4202_ruvABC_recG | 0.026411 |
| 4658_recB_recO_ruvABC-4202_ruvABC_recG | 0.113095 |
| 4659_recB_recO_recG-4202_ruvABC_recG | 0.672061 |
| 4660_recB_recO_ruvABC_recG-4202_ruvABC_recG | 0.00668 |
| TRM387_wt-4202_ruvABC_recG | 1.68E-13 |
| 4612_recB_recG-4611_recB_ruvABC | 1 |
| 4613_recB_ruvABC_recG-4611_recB_ruvABC | 0.999946 |
| 4617_recO-4611_recB_ruvABC | 5.84E-09 |
| 4618_recO_ruvABC-4611_recB_ruvABC | 1.74E-09 |
| 4619_recO_recG-4611_recB_ruvABC | 2.20E-06 |

| | |
|---|---|
| 4620_recO_ruvABC_recG-4611_recB_ruvABC | 0.028363 |
| 4621_recB_recO-4611_recB_ruvABC | 0.995672 |
| 4658_recB_recO_ruvABC-4611_recB_ruvABC | 0.872452 |
| 4659_recB_recO_recG-4611_recB_ruvABC | 0.232374 |
| 4660_recB_recO_ruvABC_recG-4611_recB_ruvABC | 0.999994 |
| TRM387_wt-4611_recB_ruvABC | 4.85E-09 |
| 4613_recB_ruvABC_recG-4612_recB_recG | 0.999412 |
| 4617_recO-4612_recB_recG | 9.31E-09 |
| 4618_recO_ruvABC-4612_recB_recG | 2.74E-09 |
| 4619_recO_recG-4612_recB_recG | 3.68E-06 |
| 4620_recO_ruvABC_recG-4612_recB_recG | 0.017755 |
| 4621_recB_recO-4612_recB_recG | 0.983568 |
| 4658_recB_recO_ruvABC-4612_recB_recG | 0.779282 |
| 4659_recB_recO_recG-4612_recB_recG | 0.16267 |
| 4660_recB_recO_ruvABC_recG-4612_recB_recG | 0.999889 |
| TRM387_wt-4612_recB_recG | 7.71E-09 |
| 4617_recO-4613_recB_ruvABC_recG | 7.60E-10 |
| 4618_recO_ruvABC-4613_recB_ruvABC_recG | 2.38E-10 |
| 4619_recO_recG-4613_recB_ruvABC_recG | 2.28E-07 |
| 4620_recO_ruvABC_recG-4613_recB_ruvABC_recG | 0.185035 |
| 4621_recB_recO-4613_recB_ruvABC_recG | 1 |
| 4658_recB_recO_ruvABC-4613_recB_ruvABC_recG | 0.999433 |
| 4659_recB_recO_recG-4613_recB_ruvABC_recG | 0.728385 |
| 4660_recB_recO_ruvABC_recG-4613_recB_ruvABC_recG | 1 |
| TRM387_wt-4613_recB_ruvABC_recG | 6.36E-10 |
| 4618_recO_ruvABC-4617_recO | 1 |
| 4619_recO_recG-4617_recO | 0.64714 |
| 4620_recO_ruvABC_recG-4617_recO | 9.82E-13 |
| 4621_recB_recO-4617_recO | 2.99E-10 |
| 4658_recB_recO_ruvABC-4617_recO | 7.14E-11 |
| 4659_recB_recO_recG-4617_recO | 6.38E-12 |
| 4660_recB_recO_ruvABC_recG-4617_recO | 1.05E-09 |
| TRM387_wt-4617_recO | 1 |
| 4619_recO_recG-4618_recO_ruvABC | 0.337233 |
| 4620_recO_ruvABC_recG-4618_recO_ruvABC | 4.32E-13 |
| 4621_recB_recO-4618_recO_ruvABC | 9.61E-11 |
| 4658_recB_recO_ruvABC-4618_recO_ruvABC | 2.38E-11 |
| 4659_recB_recO_recG-4618_recO_ruvABC | 2.35E-12 |
| 4660_recB_recO_ruvABC_recG-4618_recO_ruvABC | 3.27E-10 |
| TRM387_wt-4618_recO_ruvABC | 1 |
| 4620_recO_ruvABC_recG-4619_recO_recG | 1.10E-10 |
| 4621_recB_recO-4619_recO_recG | 8.01E-08 |

| | |
|---|---|
| 4658_recB_recO_ruvABC-4619_recO_recG | 1.60E-08 |
| 4659_recB_recO_recG-4619_recO_recG | 1.03E-09 |
| 4660_recB_recO_ruvABC_recG-4619_recO_recG | 3.27E-07 |
| TRM387_wt-4619_recO_recG | 0.597705 |
| 4621_recB_recO-4620_recO_ruvABC_recG | 0.370016 |
| 4658_recB_recO_ruvABC-4620_recO_ruvABC_recG | 0.765422 |
| 4659_recB_recO_recG-4620_recO_ruvABC_recG | 0.999725 |
| 4660_recB_recO_ruvABC_recG-4620_recO_ruvABC_recG | 0.141082 |
| TRM387_wt-4620_recO_ruvABC_recG | 8.60E-13 |
| 4658_recB_recO_ruvABC-4621_recB_recO | 0.999999 |
| 4659_recB_recO_recG-4621_recB_recO | 0.918862 |
| 4660_recB_recO_ruvABC_recG-4621_recB_recO | 1 |
| TRM387_wt-4621_recB_recO | 2.51E-10 |
| 4659_recB_recO_recG-4658_recB_recO_ruvABC | 0.998556 |
| 4660_recB_recO_ruvABC_recG-4658_recB_recO_ruvABC | 0.99777 |
| TRM387_wt-4658_recB_recO_ruvABC | 6.03E-11 |
| 4660_recB_recO_ruvABC_recG-4659_recB_recO_recG | 0.642063 |
| TRM387_wt-4659_recB_recO_recG | 5.46E-12 |
| TRM387_wt-4660_recB_recO_ruvABC_recG | 8.79E-10 |

**Table S4.** Statistical analysis of differences in the occurrence of filamentous, anucleate and regular cells in different strains after 60 min of I-*Sce*I expression (Table 2). P-values (p.adj.Chisq) are for the pairwise chi-square tests and are adjusted for multiple testing through the Bonferroni method. NA, not available.

| **Comparison strain1-strain2** | **p.adj.Chisq** |
|---|---|
| TRM387_wt-4199_ruvABC | 3.50E-177 |
| TRM387_wt-4201_recG | 1.12E-65 |
| TRM387_wt-4202_ruvABC_recG | 8.03E-202 |
| TRM387_wt-4198_recB | 2.30E-49 |
| TRM387_wt-4611_recB_ruvABC | 1.97E-26 |
| TRM387_wt-4612_recB_recG | 2.00E-40 |
| TRM387_wt-4613_recB_ruvABC_recG | 6.06E-34 |
| TRM387_wt-4617_recO | 1 |
| TRM387_wt-4618_recO_ruvABC | 2.95E-127 |
| TRM387_wt-4619_recO_recG | 1.74E-29 |
| TRM387_wt-4620_recO_ruvABC_recG | 7.78E-119 |
| TRM387_wt-4621_recB_recO | 1.54E-33 |
| TRM387_wt-4658_recB_recO_ruvABC | 3.25E-27 |
| TRM387_wt-4659_recB_recO_recG | 2.55E-31 |
| TRM387_wt-4660_recB_recO_ruvABC_recG | 1.43E-35 |
| 4199_ruvABC-4201_recG | 1.87E-41 |

| | |
|---|---|
| 4199_ruvABC-4202_ruvABC_recG | 0.00377 |
| 4199_ruvABC-4198_recB | 0 |
| 4199_ruvABC-4611_recB_ruvABC | 4.75E-284 |
| 4199_ruvABC-4612_recB_recG | 7.62E-307 |
| 4199_ruvABC-4613_recB_ruvABC_recG | 1.76E-301 |
| 4199_ruvABC-4617_recO | 2.52E-205 |
| 4199_ruvABC-4618_recO_ruvABC | 1.63E-07 |
| 4199_ruvABC-4619_recO_recG | 1.09E-71 |
| 4199_ruvABC-4620_recO_ruvABC_recG | 1.06E-12 |
| 4199_ruvABC-4621_recB_recO | 0 |
| 4199_ruvABC-4658_recB_recO_ruvABC | 1.99E-311 |
| 4199_ruvABC-4659_recB_recO_recG | 0 |
| 4199_ruvABC-4660_recB_recO_ruvABC_recG | 0 |
| 4201_recG-4202_ruvABC_recG | 1.03E-53 |
| 4201_recG-4198_recB | 3.07E-200 |
| 4201_recG-4611_recB_ruvABC | 1.99E-152 |
| 4201_recG-4612_recB_recG | 2.38E-175 |
| 4201_recG-4613_recB_ruvABC_recG | 7.72E-168 |
| 4201_recG-4617_recO | 9.40E-81 |
| 4201_recG-4618_recO_ruvABC | 2.85E-23 |
| 4201_recG-4619_recO_recG | 7.04E-07 |
| 4201_recG-4620_recO_ruvABC_recG | 5.71E-10 |
| 4201_recG-4621_recB_recO | 1.08E-182 |
| 4201_recG-4658_recB_recO_ruvABC | 2.47E-165 |
| 4201_recG-4659_recB_recO_recG | 9.25E-177 |
| 4201_recG-4660_recB_recO_ruvABC_recG | 2.55E-199 |
| 4202_ruvABC_recG-4198_recB | 0 |
| 4202_ruvABC_recG-4611_recB_ruvABC | 7.16E-313 |
| 4202_ruvABC_recG-4612_recB_recG | 0 |
| 4202_ruvABC_recG-4613_recB_ruvABC_recG | 0 |
| 4202_ruvABC_recG-4617_recO | 4.77E-230 |
| 4202_ruvABC_recG-4618_recO_ruvABC | 1.37E-22 |
| 4202_ruvABC_recG-4619_recO_recG | 1.01E-90 |
| 4202_ruvABC_recG-4620_recO_ruvABC_recG | 5.94E-17 |
| 4202_ruvABC_recG-4621_recB_recO | 0 |
| 4202_ruvABC_recG-4658_recB_recO_ruvABC | 0 |
| 4202_ruvABC_recG-4659_recB_recO_recG | 0 |
| 4202_ruvABC_recG-4660_recB_recO_ruvABC_recG | 0 |
| 4198_recB-4611_recB_ruvABC | 1.36E-05 |
| 4198_recB-4612_recB_recG | NA |
| 4198_recB-4613_recB_ruvABC_recG | NA |
| 4198_recB-4617_recO | 1.00E-45 |
| 4198_recB-4618_recO_ruvABC | 8.39E-274 |

| | |
|---|---|
| 4198_recB-4619_recO_recG | 3.44E-137 |
| 4198_recB-4620_recO_ruvABC_recG | 2.40E-273 |
| 4198_recB-4621_recB_recO | 1.02E-07 |
| 4198_recB-4658_recB_recO_ruvABC | 9.27E-12 |
| 4198_recB-4659_recB_recO_recG | 4.25E-14 |
| 4198_recB-4660_recB_recO_ruvABC_recG | 3.18E-14 |
| 4611_recB_ruvABC-4612_recB_recG | 0.00367 |
| 4611_recB_ruvABC-4613_recB_ruvABC_recG | 1 |
| 4611_recB_ruvABC-4617_recO | 2.63E-23 |
| 4611_recB_ruvABC-4618_recO_ruvABC | 1.92E-223 |
| 4611_recB_ruvABC-4619_recO_recG | 1.44E-98 |
| 4611_recB_ruvABC-4620_recO_ruvABC_recG | 4.41E-219 |
| 4611_recB_ruvABC-4621_recB_recO | 1 |
| 4611_recB_ruvABC-4658_recB_recO_ruvABC | 1 |
| 4611_recB_ruvABC-4659_recB_recO_recG | 0.456 |
| 4611_recB_ruvABC-4660_recB_recO_ruvABC_recG | 1 |
| 4612_recB_recG-4613_recB_ruvABC_recG | NA |
| 4612_recB_recG-4617_recO | 3.45E-37 |
| 4612_recB_recG-4618_recO_ruvABC | 6.98E-244 |
| 4612_recB_recG-4619_recO_recG | 3.58E-118 |
| 4612_recB_recG-4620_recO_ruvABC_recG | 2.05E-243 |
| 4612_recB_recG-4621_recB_recO | 0.000105 |
| 4612_recB_recG-4658_recB_recO_ruvABC | 5.41E-08 |
| 4612_recB_recG-4659_recB_recO_recG | 7.52E-10 |
| 4612_recB_recG-4660_recB_recO_ruvABC_recG | 6.18E-10 |
| 4613_recB_ruvABC_recG-4617_recO | 1.22E-30 |
| 4613_recB_ruvABC_recG-4618_recO_ruvABC | 5.49E-239 |
| 4613_recB_ruvABC_recG-4619_recO_recG | 5.21E-111 |
| 4613_recB_ruvABC_recG-4620_recO_ruvABC_recG | 2.26E-236 |
| 4613_recB_ruvABC_recG-4621_recB_recO | 1 |
| 4613_recB_ruvABC_recG-4658_recB_recO_ruvABC | 0.0073 |
| 4613_recB_ruvABC_recG-4659_recB_recO_recG | 0.000508 |
| 4613_recB_ruvABC_recG-4660_recB_recO_ruvABC_recG | 0.000397 |
| 4617_recO-4618_recO_ruvABC | 6.54E-152 |
| 4617_recO-4619_recO_recG | 1.17E-38 |
| 4617_recO-4620_recO_ruvABC_recG | 6.74E-140 |
| 4617_recO-4621_recB_recO | 1.64E-29 |
| 4617_recO-4658_recB_recO_ruvABC | 1.66E-23 |
| 4617_recO-4659_recB_recO_recG | 4.32E-27 |
| 4617_recO-4660_recB_recO_ruvABC_recG | 8.54E-31 |
| 4618_recO_ruvABC-4619_recO_recG | 5.86E-40 |
| 4618_recO_ruvABC-4620_recO_ruvABC_recG | 5.91E-13 |
| 4618_recO_ruvABC-4621_recB_recO | 9.44E-268 |

| | |
|---|---|
| 4618_recO_ruvABC-4658_recB_recO_ruvABC | 7.61E-249 |
| 4618_recO_ruvABC-4659_recB_recO_recG | 3.99E-268 |
| 4618_recO_ruvABC-4660_recB_recO_ruvABC_recG | 1.16E-301 |
| 4619_recO_recG-4620_recO_ruvABC_recG | 4.07E-34 |
| 4619_recO_recG-4621_recB_recO | 1.65E-120 |
| 4619_recO_recG-4658_recB_recO_ruvABC | 2.48E-107 |
| 4619_recO_recG-4659_recB_recO_recG | 1.61E-116 |
| 4619_recO_recG-4660_recB_recO_ruvABC_recG | 1.04E-132 |
| 4620_recO_ruvABC_recG-4621_recB_recO | 2.54E-258 |
| 4620_recO_ruvABC_recG-4658_recB_recO_ruvABC | 6.59E-238 |
| 4620_recO_ruvABC_recG-4659_recB_recO_recG | 5.62E-253 |
| 4620_recO_ruvABC_recG-4660_recB_recO_ruvABC_recG | 2.06E-282 |
| 4621_recB_recO-4658_recB_recO_ruvABC | 1 |
| 4621_recB_recO-4659_recB_recO_recG | 1 |
| 4621_recB_recO-4660_recB_recO_ruvABC_recG | 1 |
| 4658_recB_recO_ruvABC-4659_recB_recO_recG | 1 |
| 4658_recB_recO_ruvABC-4660_recB_recO_ruvABC_recG | 1 |
| 4659_recB_recO_recG-4660_recB_recO_ruvABC_recG | 1 |

**Table S5.** *E. coli* strains divided into groups (Tukey HSD test, alpha=0.05) based on the data analysed on γ-survival at 200 Gy (see also Figure 4). Groups labeled with identical letters don't differ significantly in mean log survival rates. The group labels are ordered by the decreasing survival rates.

| **Strain/genotype** | **Group** |
|---|---|
| 2629_wt | a |
| 3188_ruvABC | ab |
| 3196_recG | ab |
| 3545_recO | bc |
| 3600_recO_ruvABC | cd |
| 3601_recO_recG | d |
| 3183_recB | e |
| 3625_recB_ruvABC | e |
| 3682_recB_recG | f |
| 4134_recB_ruvABC_recG | f |
| 3599_recB_recO | g |
| 3602_recB_recO_ruvABC | g |
| 3605_recB_recO_ruvABC_recG | g |
| 3604_recO_ruvABC_recG | g |
| 3603_recB_recO_recG | gh |
| 3610_ruvABC_recG | h |

**Table S6.** Statistical analysis of differences in survival after 200 Gy of γ-irradiation (see also Figure 4) presented as pairwise p-values (p adj) from the Tukey HSD test, which controls for multiple testing.

| **Comparison strain1-strain2** | **p adj** |
|---|---|
| 3183_recB-2629_wt | 1,1001E-12 |
| 3196_recG-2629_wt | 0,32165629 |
| 3545_recO-2629_wt | 0,00041617 |
| 3599_recB_recO-2629_wt | 1,1001E-12 |
| 3600_recO_ruvABC-2629_wt | 5,0985E-08 |
| 3601_recO_recG-2629_wt | 2,4518E-12 |
| 3602_recB_recO_ruvABC-2629_wt | 1,1001E-12 |
| 3603_recB_recO_recG-2629_wt | 1,1001E-12 |
| 3604_recO_ruvABC_recG-2629_wt | 1,1001E-12 |
| 3605_recB_recO_ruvABC_recG-2629_wt | 1,1001E-12 |
| 3610_ruvABC_recG-2629_wt | 1,1001E-12 |
| 3625_recB_ruvABC-2629_wt | 1,1001E-12 |
| 3682_recB_recG-2629_wt | 1,1001E-12 |
| 4134_recB_ruvABC_recG-2629_wt | 1,1001E-12 |
| 3188_ruvABC-3183_recB | 1,1001E-12 |
| 3196_recG-3183_recB | 1,1001E-12 |
| 3545_recO-3183_recB | 1,1001E-12 |
| 3599_recB_recO-3183_recB | 1,4956E-11 |
| 3600_recO_ruvABC-3183_recB | 1,1002E-12 |
| 3601_recO_recG-3183_recB | 1,1263E-12 |
| 3602_recB_recO_ruvABC-3183_recB | 8,4797E-12 |
| 3603_recB_recO_recG-3183_recB | 1,103E-12 |
| 3604_recO_ruvABC_recG-3183_recB | 1,2135E-12 |
| 3605_recB_recO_ruvABC_recG-3183_recB | 1,5792E-12 |
| 3610_ruvABC_recG-3183_recB | 1,1001E-12 |
| 3625_recB_ruvABC-3183_recB | 1 |
| 3682_recB_recG-3183_recB | 0,00119292 |
| 4134_recB_ruvABC_recG-3183_recB | 3,5438E-06 |
| 3196_recG-3188_ruvABC | 1 |
| 3545_recO-3188_ruvABC | 0,49711558 |
| 3599_recB_recO-3188_ruvABC | 1,1001E-12 |
| 3600_recO_ruvABC-3188_ruvABC | 0,00114002 |
| 3601_recO_recG-3188_ruvABC | 4,5958E-08 |
| 3602_recB_recO_ruvABC-3188_ruvABC | 1,1001E-12 |
| 3603_recB_recO_recG-3188_ruvABC | 1,1001E-12 |
| 3604_recO_ruvABC_recG-3188_ruvABC | 1,1001E-12 |
| 3605_recB_recO_ruvABC_recG-3188_ruvABC | 1,1001E-12 |
| 3610_ruvABC_recG-3188_ruvABC | 1,1001E-12 |
| 3625_recB_ruvABC-3188_ruvABC | 1,1001E-12 |
| 3682_recB_recG-3188_ruvABC | 1,1001E-12 |
| 4134_recB_ruvABC_recG-3188_ruvABC | 1,1001E-12 |
| 3545_recO-3196_recG | 0,67156974 |
| 3599_recB_recO-3196_recG | 1,1001E-12 |
| 3600_recO_ruvABC-3196_recG | 0,00252715 |
| 3601_recO_recG-3196_recG | 1,0619E-07 |
| 3602_recB_recO_ruvABC-3196_recG | 1,1001E-12 |

| | |
|---|---|
| 3603_recB_recO_recG-3196_recG | 1,1001E-12 |
| 3604_recO_ruvABC_recG-3196_recG | 1,1001E-12 |
| 3605_recB_recO_ruvABC_recG-3196_recG | 1,1001E-12 |
| 3610_ruvABC_recG-3196_recG | 1,1001E-12 |
| 3625_recB_ruvABC-3196_recG | 1,1001E-12 |
| 3682_recB_recG-3196_recG | 1,1001E-12 |
| 4134_recB_ruvABC_recG-3196_recG | 1,1001E-12 |
| 3599_recB_recO-3545_recO | 1,1001E-12 |
| 3600_recO_ruvABC-3545_recO | 0,51701111 |
| 3601_recO_recG-3545_recO | 0,00016229 |
| 3602_recB_recO_ruvABC-3545_recO | 1,1001E-12 |
| 3603_recB_recO_recG-3545_recO | 1,1001E-12 |
| 3604_recO_ruvABC_recG-3545_recO | 1,1001E-12 |
| 3605_recB_recO_ruvABC_recG-3545_recO | 1,1001E-12 |
| 3610_ruvABC_recG-3545_recO | 1,1001E-12 |
| 3625_recB_ruvABC-3545_recO | 1,1001E-12 |
| 3682_recB_recG-3545_recO | 1,1001E-12 |
| 4134_recB_ruvABC_recG-3545_recO | 1,1001E-12 |
| 3600_recO_ruvABC-3599_recB_recO | 1,1001E-12 |
| 3601_recO_recG-3599_recB_recO | 1,1001E-12 |
| 3602_recB_recO_ruvABC-3599_recB_recO | 1 |
| 3603_recB_recO_recG-3599_recB_recO | 0,10921846 |
| 3604_recO_ruvABC_recG-3599_recB_recO | 0,96116862 |
| 3605_recB_recO_ruvABC_recG-3599_recB_recO | 0,99915218 |
| 3610_ruvABC_recG-3599_recB_recO | 3,2468E-05 |
| 3625_recB_ruvABC-3599_recB_recO | 1,3055E-09 |
| 3682_recB_recG-3599_recB_recO | 0,00065138 |
| 4134_recB_ruvABC_recG-3599_recB_recO | 6,4711E-05 |
| 3601_recO_recG-3600_recO_ruvABC | 0,17238577 |
| 3602_recB_recO_ruvABC-3600_recO_ruvABC | 1,1001E-12 |
| 3603_recB_recO_recG-3600_recO_ruvABC | 1,1001E-12 |
| 3604_recO_ruvABC_recG-3600_recO_ruvABC | 1,1001E-12 |
| 3605_recB_recO_ruvABC_recG-3600_recO_ruvABC | 1,1001E-12 |
| 3610_ruvABC_recG-3600_recO_ruvABC | 1,1001E-12 |
| 3625_recB_ruvABC-3600_recO_ruvABC | 1,1008E-12 |
| 3682_recB_recG-3600_recO_ruvABC | 1,1001E-12 |
| 4134_recB_ruvABC_recG-3600_recO_ruvABC | 1,1001E-12 |
| 3602_recB_recO_ruvABC-3601_recO_recG | 1,1001E-12 |
| 3603_recB_recO_recG-3601_recO_recG | 1,1001E-12 |
| 3604_recO_ruvABC_recG-3601_recO_recG | 1,1001E-12 |
| 3605_recB_recO_ruvABC_recG-3601_recO_recG | 1,1001E-12 |
| 3610_ruvABC_recG-3601_recO_recG | 1,1001E-12 |
| 3625_recB_ruvABC-3601_recO_recG | 1,3546E-12 |
| 3682_recB_recG-3601_recO_recG | 1,1005E-12 |
| 4134_recB_ruvABC_recG-3601_recO_recG | 1,1001E-12 |
| 3603_recB_recO_recG-3602_recB_recO_ruvABC | 0,16689445 |
| 3604_recO_ruvABC_recG-3602_recB_recO_ruvABC | 0,9874719 |
| 3605_recB_recO_ruvABC_recG-3602_recB_recO_ruvABC | 0,99991888 |
| 3610_ruvABC_recG-3602_recB_recO_ruvABC | 6,6079E-05 |
| 3625_recB_ruvABC-3602_recB_recO_ruvABC | 7,1406E-10 |

| | |
|---|---|
| 3682_recB_recG-3602_recB_recO_ruvABC | 0,00035136 |
| 4134_recB_ruvABC_recG-3602_recB_recO_ruvABC | 3,0058E-05 |
| 3604_recO_ruvABC_recG-3603_recB_recO_recG | 0,93043756 |
| 3605_recB_recO_ruvABC_recG-3603_recB_recO_recG | 0,67093631 |
| 3610_ruvABC_recG-3603_recB_recO_recG | 0,59003804 |
| 3625_recB_ruvABC-3603_recB_recO_recG | 1,2055E-12 |
| 3682_recB_recG-3603_recB_recO_recG | 1,3191E-08 |
| 4134_recB_ruvABC_recG-3603_recB_recO_recG | 1,6719E-10 |
| 3605_recB_recO_ruvABC_recG-3604_recO_ruvABC_recG | 0,99999994 |
| 3610_ruvABC_recG-3604_recO_ruvABC_recG | 0,00877785 |
| 3625_recB_ruvABC-3604_recO_ruvABC_recG | 1,1628E-11 |
| 3682_recB_recG-3604_recO_ruvABC_recG | 3,6069E-06 |
| 4134_recB_ruvABC_recG-3604_recO_ruvABC_recG | 1,1636E-07 |
| 3610_ruvABC_recG-3605_recB_recO_ruvABC_recG | 0,00155884 |
| 3625_recB_ruvABC-3605_recB_recO_ruvABC_recG | 4,9202E-11 |
| 3682_recB_recG-3605_recB_recO_ruvABC_recG | 1,9731E-05 |
| 4134_recB_ruvABC_recG-3605_recB_recO_ruvABC_recG | 8,9137E-07 |
| 3625_recB_ruvABC-3610_ruvABC_recG | 1,1005E-12 |
| 3682_recB_recG-3610_ruvABC_recG | 1,7021E-12 |
| 4134_recB_ruvABC_recG-3610_ruvABC_recG | 1,1026E-12 |
| 3682_recB_recG-3625_recB_ruvABC | 0,01694308 |
| 4134_recB_ruvABC_recG-3625_recB_ruvABC | 0,00049422 |
| 4134_recB_ruvABC_recG-3682_recB_recG | 1 |

**Table S7.** Statistical analysis of differences in the occurrence of filamentous, anucleate and regular cells in different strains after UV-dose of 5 J/$m^2$ (Table 3). P-values (p.adj.Chisq) are for the pairwise chi-square tests and are adjusted for multiple testing through the Bonferroni method.

| **Comparison strain1:strain2** | **p.adj.Chisq** |
|---|---|
| 2629_wt : 3188_ruvABC | 0 |
| 2629_wt : 3196_recG | 9,86E-136 |
| 2629_wt : 3610_ruvABC_recG | 0 |
| 2629_wt : 3182_recB | 2,84E-14 |
| 2629_wt : 3625_recB_ruvABC | 1,38E-206 |
| 2629_wt : 3626_recB_recG | 7,12E-16 |
| 2629_wt : 4134_recB_ruvABC_recG | 1,86E-65 |
| 2629_wt : 3545_recO | 8,16E-304 |
| 2629_wt : 3600_recO_ruvABC | 0 |
| 2629_wt : 3601_recO_recG | 0 |
| 2629_wt : 3604_recO_ruvABC_recG | 0 |
| 2629_wt : 3599_recB_recO | 3,88E-45 |
| 2629_wt : 3602_recB_recO_ruvABC | 1,39E-44 |
| 2629_wt : 3603_recB_recO_recG | 1,54E-35 |
| 2629_wt : 3605_recB_recO_ruvABC_recG | 7,8E-33 |
| 3188_ruvABC : 3196_recG | 4,21E-194 |
| 3188_ruvABC : 3610_ruvABC_recG | 4,9E-15 |
| 3188_ruvABC : 3182_recB | 0 |
| 3188_ruvABC : 3625_recB_ruvABC | 1,51E-83 |
| 3188_ruvABC : 3626_recB_recG | 0 |

| | |
|---|---|
| 3188_ruvABC : 4134_recB_ruvABC_recG | 0 |
| 3188_ruvABC : 3545_recO | 1,96E-163 |
| 3188_ruvABC : 3600_recO_ruvABC | 1,17E-86 |
| 3188_ruvABC : 3601_recO_recG | 7,34E-146 |
| 3188_ruvABC : 3604_recO_ruvABC_recG | 1,79E-20 |
| 3188_ruvABC : 3599_recB_recO | 3.07000000023002e-314 |
| 3188_ruvABC : 3602_recB_recO_ruvABC | 0 |
| 3188_ruvABC : 3603_recB_recO_recG | 0 |
| 3188_ruvABC : 3605_recB_recO_ruvABC_recG | 0 |
| 3196_recG : 3610_ruvABC_recG | 1,94E-232 |
| 3196_recG : 3182_recB | 1,16E-182 |
| 3196_recG : 3625_recB_ruvABC | 1,3E-32 |
| 3196_recG : 3626_recB_recG | 2,7E-114 |
| 3196_recG : 4134_recB_ruvABC_recG | 1,57E-34 |
| 3196_recG : 3545_recO | 2,48E-59 |
| 3196_recG : 3600_recO_ruvABC | 4,58E-104 |
| 3196_recG : 3601_recO_recG | 2,62E-86 |
| 3196_recG : 3604_recO_ruvABC_recG | 1,43E-115 |
| 3196_recG : 3599_recB_recO | 2,9E-51 |
| 3196_recG : 3602_recB_recO_ruvABC | 6,53E-55 |
| 3196_recG : 3603_recB_recO_recG | 1,08E-78 |
| 3196_recG : 3605_recB_recO_ruvABC_recG | 7,32E-98 |
| 3610_ruvABC_recG : 3182_recB | 0 |
| 3610_ruvABC_recG : 3625_recB_ruvABC | 4,58E-127 |
| 3610_ruvABC_recG : 3626_recB_recG | 0 |
| 3610_ruvABC_recG : 4134_recB_ruvABC_recG | 0 |
| 3610_ruvABC_recG : 3545_recO | 3,4E-140 |
| 3610_ruvABC_recG : 3600_recO_ruvABC | 5,87E-56 |
| 3610_ruvABC_recG : 3601_recO_recG | 4,25E-113 |
| 3610_ruvABC_recG : 3604_recO_ruvABC_recG | 7,04E-10 |
| 3610_ruvABC_recG : 3599_recB_recO | 0 |
| 3610_ruvABC_recG : 3602_recB_recO_ruvABC | 0 |
| 3610_ruvABC_recG : 3603_recB_recO_recG | 0 |
| 3610_ruvABC_recG : 3605_recB_recO_ruvABC_recG | 0 |
| 3182_recB : 3625_recB_ruvABC | 3.19000000963269e-316 |
| 3182_recB : 3626_recB_recG | 1 |
| 3182_recB : 4134_recB_ruvABC_recG | 3,05E-95 |
| 3182_recB : 3545_recO | 0 |
| 3182_recB : 3600_recO_ruvABC | 0 |
| 3182_recB : 3601_recO_recG | 0 |
| 3182_recB : 3604_recO_ruvABC_recG | 0 |
| 3182_recB : 3599_recB_recO | 8,69E-25 |
| 3182_recB : 3602_recB_recO_ruvABC | 5,5E-30 |
| 3182_recB : 3603_recB_recO_recG | 1,2E-16 |
| 3182_recB : 3605_recB_recO_ruvABC_recG | 2,62E-13 |
| 3625_recB_ruvABC : 3626_recB_recG | 4,46E-206 |
| 3625_recB_ruvABC : 4134_recB_ruvABC_recG | 2,39E-93 |
| 3625_recB_ruvABC : 3545_recO | 1,94E-74 |
| 3625_recB_ruvABC : 3600_recO_ruvABC | 8,82E-78 |
| 3625_recB_ruvABC : 3601_recO_recG | 4,98E-95 |

| | |
|---|---|
| 3625_recB_ruvABC : 3604_recO_ruvABC_recG | 2,5E-53 |
| 3625_recB_ruvABC : 3599_recB_recO | 1,31E-120 |
| 3625_recB_ruvABC : 3602_recB_recO_ruvABC | 6,49E-134 |
| 3625_recB_ruvABC : 3603_recB_recO_recG | 5,2E-170 |
| 3625_recB_ruvABC : 3605_recB_recO_ruvABC_recG | 7,46E-204 |
| 3626_recB_recG : 4134_recB_ruvABC_recG | 2,54E-56 |
| 3626_recB_recG : 3545_recO | 1,5E-278 |
| 3626_recB_recG : 3600_recO_ruvABC | 0 |
| 3626_recB_recG : 3601_recO_recG | 9.47999999999849e-312 |
| 3626_recB_recG : 3604_recO_ruvABC_recG | 0 |
| 3626_recB_recG : 3599_recB_recO | 2,3E-12 |
| 3626_recB_recG : 3602_recB_recO_ruvABC | 4,25E-15 |
| 3626_recB_recG : 3603_recB_recO_recG | 9,94E-07 |
| 3626_recB_recG : 3605_recB_recO_ruvABC_recG | 0,000136 |
| 4134_recB_ruvABC_recG : 3545_recO | 1,24E-209 |
| 4134_recB_ruvABC_recG : 3600_recO_ruvABC | 1,01E-269 |
| 4134_recB_ruvABC_recG : 3601_recO_recG | 2,56E-248 |
| 4134_recB_ruvABC_recG : 3604_recO_ruvABC_recG | 1,03E-251 |
| 4134_recB_ruvABC_recG : 3599_recB_recO | 4,5E-40 |
| 4134_recB_ruvABC_recG : 3602_recB_recO_ruvABC | 1,16E-40 |
| 4134_recB_ruvABC_recG : 3603_recB_recO_recG | 1,46E-49 |
| 4134_recB_ruvABC_recG : 3605_recB_recO_ruvABC_recG | 2,42E-59 |
| 3545_recO : 3600_recO_ruvABC | 6,65E-19 |
| 3545_recO : 3601_recO_recG | 0,00211 |
| 3545_recO : 3604_recO_ruvABC_recG | 2,53E-65 |
| 3545_recO : 3599_recB_recO | 5,35E-147 |
| 3545_recO : 3602_recB_recO_ruvABC | 2,11E-160 |
| 3545_recO : 3603_recB_recO_recG | 1,33E-206 |
| 3545_recO : 3605_recB_recO_ruvABC_recG | 5,95E-243 |
| 3600_recO_ruvABC : 3601_recO_recG | 3,91E-10 |
| 3600_recO_ruvABC : 3604_recO_ruvABC_recG | 2,1E-19 |
| 3600_recO_ruvABC : 3599_recB_recO | 1,08E-218 |
| 3600_recO_ruvABC : 3602_recB_recO_ruvABC | 2,72E-236 |
| 3600_recO_ruvABC : 3603_recB_recO_recG | 6,74E-289 |
| 3600_recO_ruvABC : 3605_recB_recO_ruvABC_recG | 0 |
| 3601_recO_recG : 3604_recO_ruvABC_recG | 2,4E-55 |
| 3601_recO_recG : 3599_recB_recO | 4,34E-175 |
| 3601_recO_recG : 3602_recB_recO_ruvABC | 4,2E-189 |
| 3601_recO_recG : 3603_recB_recO_recG | 4,39E-237 |
| 3601_recO_recG : 3605_recB_recO_ruvABC_recG | 6,07E-275 |
| 3604_recO_ruvABC_recG : 3599_recB_recO | 4,94E-235 |
| 3604_recO_ruvABC_recG : 3602_recB_recO_ruvABC | 2,78E-255 |
| 3604_recO_ruvABC_recG : 3603_recB_recO_recG | 1,16E-307 |
| 3604_recO_ruvABC_recG : 3605_recB_recO_ruvABC_recG | 0 |
| 3599_recB_recO : 3602_recB_recO_ruvABC | 1 |
| 3599_recB_recO : 3603_recB_recO_recG | 1 |
| 3599_recB_recO : 3605_recB_recO_ruvABC_recG | 0,403 |
| 3602_recB_recO_ruvABC : 3603_recB_recO_recG | 0,569 |
| 3602_recB_recO_ruvABC : 3605_recB_recO_ruvABC_recG | 0,00454 |
| 3603_recB_recO_recG : 3605_recB_recO_ruvABC_recG | 1 |

**Table S8.** Statistical analysis of differences in the occurrence of filamentous, anucleate and regular cells in different strains after γ-dose of 100 Gy (Table 3). P-values (p.adj.Chisq) are for the pairwise chi-square tests and are adjusted for multiple testing through the Bonferroni method.

| **Comparison strain1:strain2** | **p.adj.Chisq** |
|---|---|
| 2629_wt : 3188_ruvABC | 1,63E-252 |
| 2629_wt : 3196_recG | 5,41E-44 |
| 2629_wt : 3610_ruvABC_recG | 0 |
| 2629_wt : 3182_recB | 0,00000092 |
| 2629_wt : 3625_recB_ruvABC | 1,86E-29 |
| 2629_wt : 3626_recB_recG | 7,42E-07 |
| 2629_wt : 4134_recB_ruvABC_recG | 0,0000844 |
| 2629_wt : 3545_recO | 5,99E-15 |
| 2629_wt : 3600_recO_ruvABC | 6,91E-280 |
| 2629_wt : 3601_recO_recG | 3,18E-56 |
| 2629_wt : 3604_recO_ruvABC_recG | 5,73E-285 |
| 2629_wt : 3599_recB_recO | 1,25E-19 |
| 2629_wt : 3602_recB_recO_ruvABC | 9,91E-20 |
| 2629_wt : 3603_recB_recO_recG | 2,88E-21 |
| 2629_wt : 3605_recB_recO_ruvABC_recG | 1,8E-19 |
| 3188_ruvABC : 3196_recG | 1,72E-162 |
| 3188_ruvABC : 3610_ruvABC_recG | 1,87E-54 |
| 3188_ruvABC : 3182_recB | 7,8E-292 |
| 3188_ruvABC : 3625_recB_ruvABC | 7,14E-127 |
| 3188_ruvABC : 3626_recB_recG | 0 |
| 3188_ruvABC : 4134_recB_ruvABC_recG | 3,38E-259 |
| 3188_ruvABC : 3545_recO | 5,63E-168 |
| 3188_ruvABC : 3600_recO_ruvABC | 0,376 |
| 3188_ruvABC : 3601_recO_recG | 6,52E-100 |
| 3188_ruvABC : 3604_recO_ruvABC_recG | 2,25E-14 |
| 3188_ruvABC : 3599_recB_recO | 6,27E-205 |
| 3188_ruvABC : 3602_recB_recO_ruvABC | 1,6E-239 |
| 3188_ruvABC : 3603_recB_recO_recG | 1,37E-230 |
| 3188_ruvABC : 3605_recB_recO_ruvABC_recG | 5,83E-300 |
| 3196_recG : 3610_ruvABC_recG | 1,58E-250 |
| 3196_recG : 3182_recB | 1,54E-65 |
| 3196_recG : 3625_recB_ruvABC | 9,28E-15 |
| 3196_recG : 3626_recB_recG | 3,17E-78 |
| 3196_recG : 4134_recB_ruvABC_recG | 3,26E-37 |
| 3196_recG : 3545_recO | 4,22E-25 |
| 3196_recG : 3600_recO_ruvABC | 9,99E-180 |
| 3196_recG : 3601_recO_recG | 1,63E-15 |
| 3196_recG : 3604_recO_ruvABC_recG | 1,72E-161 |
| 3196_recG : 3599_recB_recO | 5,09E-14 |
| 3196_recG : 3602_recB_recO_ruvABC | 2,38E-19 |
| 3196_recG : 3603_recB_recO_recG | 3,62E-17 |
| 3196_recG : 3605_recB_recO_ruvABC_recG | 1,79E-25 |
| 3610_ruvABC_recG : 3182_recB | 0 |
| 3610_ruvABC_recG : 3625_recB_ruvABC | 1,47E-239 |
| 3610_ruvABC_recG : 3626_recB_recG | 0 |

| | |
|---|---|
| 3610_ruvABC_recG : 4134_recB_ruvABC_recG | 0 |
| 3610_ruvABC_recG : 3545_recO | 5,96E-297 |
| 3610_ruvABC_recG : 3600_recO_ruvABC | 1,31E-35 |
| 3610_ruvABC_recG : 3601_recO_recG | 1,54E-195 |
| 3610_ruvABC_recG : 3604_recO_ruvABC_recG | 2,13E-19 |
| 3610_ruvABC_recG : 3599_recB_recO | 1.2400010172759e-318 |
| 3610_ruvABC_recG : 3602_recB_recO_ruvABC | 0 |
| 3610_ruvABC_recG : 3603_recB_recO_recG | 0 |
| 3610_ruvABC_recG : 3605_recB_recO_ruvABC_recG | 0 |
| 3182_recB : 3625_recB_ruvABC | 1,42E-54 |
| 3182_recB : 3626_recB_recG | 1 |
| 3182_recB : 4134_recB_ruvABC_recG | 0,000301 |
| 3182_recB : 3545_recO | 6,83E-36 |
| 3182_recB : 3600_recO_ruvABC | 1.20996676666521e-320 |
| 3182_recB : 3601_recO_recG | 8,85E-86 |
| 3182_recB : 3604_recO_ruvABC_recG | 0 |
| 3182_recB : 3599_recB_recO | 1,04E-21 |
| 3182_recB : 3602_recB_recO_ruvABC | NA |
| 3182_recB : 3603_recB_recO_recG | NA |
| 3182_recB : 3605_recB_recO_ruvABC_recG | 1,63E-18 |
| 3625_recB_ruvABC : 3626_recB_recG | 2,64E-64 |
| 3625_recB_ruvABC : 4134_recB_ruvABC_recG | 8,89E-36 |
| 3625_recB_ruvABC : 3545_recO | 0,0365 |
| 3625_recB_ruvABC : 3600_recO_ruvABC | 2,5E-147 |
| 3625_recB_ruvABC : 3601_recO_recG | 0,00701 |
| 3625_recB_ruvABC : 3604_recO_ruvABC_recG | 1,98E-144 |
| 3625_recB_ruvABC : 3599_recB_recO | 3,52E-25 |
| 3625_recB_ruvABC : 3602_recB_recO_ruvABC | 1,45E-31 |
| 3625_recB_ruvABC : 3603_recB_recO_recG | 3,71E-30 |
| 3625_recB_ruvABC : 3605_recB_recO_ruvABC_recG | 3,16E-41 |
| 3626_recB_recG : 4134_recB_ruvABC_recG | 0,00000364 |
| 3626_recB_recG : 3545_recO | 3,66E-42 |
| 3626_recB_recG : 3600_recO_ruvABC | 0 |
| 3626_recB_recG : 3601_recO_recG | 7,79E-101 |
| 3626_recB_recG : 3604_recO_ruvABC_recG | 0 |
| 3626_recB_recG : 3599_recB_recO | 4,26E-29 |
| 3626_recB_recG : 3602_recB_recO_ruvABC | 1,72E-27 |
| 3626_recB_recG : 3603_recB_recO_recG | 4,63E-30 |
| 3626_recB_recG : 3605_recB_recO_ruvABC_recG | 1,5E-25 |
| 4134_recB_ruvABC_recG : 3545_recO | 9,09E-24 |
| 4134_recB_ruvABC_recG : 3600_recO_ruvABC | 1,19E-285 |
| 4134_recB_ruvABC_recG : 3601_recO_recG | 5,91E-60 |
| 4134_recB_ruvABC_recG : 3604_recO_ruvABC_recG | 1,52E-285 |
| 4134_recB_ruvABC_recG : 3599_recB_recO | 9,45E-07 |
| 4134_recB_ruvABC_recG : 3602_recB_recO_ruvABC | 0,00000454 |
| 4134_recB_ruvABC_recG : 3603_recB_recO_recG | 2,47E-07 |
| 4134_recB_ruvABC_recG : 3605_recB_recO_ruvABC_recG | 0,000199 |
| 3545_recO : 3600_recO_ruvABC | 1,17E-191 |
| 3545_recO : 3601_recO_recG | 1,21E-14 |
| 3545_recO : 3604_recO_ruvABC_recG | 2,59E-192 |

| | |
|---|---|
| 3545_recO : 3599_recB_recO | 1,64E-23 |
| 3545_recO : 3602_recB_recO_ruvABC | 8,25E-28 |
| 3545_recO : 3603_recB_recO_recG | 1,51E-27 |
| 3545_recO : 3605_recB_recO_ruvABC_recG | 1,46E-34 |
| 3600_recO_ruvABC : 3601_recO_recG | 3,58E-117 |
| 3600_recO_ruvABC : 3604_recO_ruvABC_recG | 2,14E-07 |
| 3600_recO_ruvABC : 3599_recB_recO | 6,87E-227 |
| 3600_recO_ruvABC : 3602_recB_recO_ruvABC | 4,73E-263 |
| 3600_recO_ruvABC : 3603_recB_recO_recG | 1,59E-253 |
| 3600_recO_ruvABC : 3605_recB_recO_ruvABC_recG | 0 |
| 3601_recO_recG : 3604_recO_ruvABC_recG | 7,11E-109 |
| 3601_recO_recG : 3599_recB_recO | 2,28E-37 |
| 3601_recO_recG : 3602_recB_recO_ruvABC | 4,79E-47 |
| 3601_recO_recG : 3603_recB_recO_recG | 3,09E-44 |
| 3601_recO_recG : 3605_recB_recO_ruvABC_recG | 5,67E-62 |
| 3604_recO_ruvABC_recG : 3599_recB_recO | 6,2E-219 |
| 3604_recO_ruvABC_recG : 3602_recB_recO_ruvABC | 1,77E-252 |
| 3604_recO_ruvABC_recG : 3603_recB_recO_recG | 9,95E-243 |
| 3604_recO_ruvABC_recG : 3605_recB_recO_ruvABC_recG | 2.09e-309 |
| 3599_recB_recO : 3602_recB_recO_ruvABC | 1 |
| 3599_recB_recO : 3603_recB_recO_recG | 1 |
| 3599_recB_recO : 3605_recB_recO_ruvABC_recG | 1 |
| 3602_recB_recO_ruvABC : 3603_recB_recO_recG | NA |
| 3602_recB_recO_ruvABC : 3605_recB_recO_ruvABC_recG | 1 |
| 3603_recB_recO_recG : 3605_recB_recO_ruvABC_recG | 1 |

**Table S9.** *E. coli* strains divided into groups (Tukey HSD test, alpha=0.05) based on the data on UV-survival at 50 J/m$^2$ (see also Figure 9). Groups labeled with identical letters don't differ significantly in mean log survival rates. The group labels are ordered by the decreasing survival rates

| **Strain/genotype** | **Group** |
|---|---|
| 2629_wt | a |
| 3196_recG | b |
| 3545_recO | bc |
| 3625_recB_ruvABC | bcd |
| 3188_ruvABC | bcd |
| 3601_recO_recG | cd |
| 3183_recB | de |
| 3600_recO_ruvABC | e |
| 3626_recB_recG | f |
| 4134_recB_ruvABC_recG | g |
| 3602_recB_recO_ruvABC | g |
| 3599__recB_recO | g |
| 3605_recB_recO_ruvABC_recG | g |
| 3603_recB_recO_recG | gh |
| 3610_ruvABC_recG | hi |
| 3604_recO_ruvABC_recG | i |

**Table S10.** Statistical analysis of differences in survival after 50 J/m$^2$ of UV (see also Figure 9) presented as pairwise p-values (p adj) from the Tukey HSD test, which controls for multiple testing.

| **Comparison strain1-strain2** | **p adj** |
|---|---|
| 3183_recB-2629_wt | 1,8079E-12 |
| 3188_ruvABC-2629_wt | 2,0665E-08 |
| 3196_recG-2629_wt | 5,4367E-06 |
| 3545_recO-2629_wt | 4,4094E-07 |
| 3599__recB_recO-2629_wt | 0 |
| 3600_recO_ruvABC-2629_wt | 0 |
| 3601_recO_recG-2629_wt | 9,0727E-11 |
| 3602_recB_recO_ruvABC-2629_wt | 0 |
| 3603_recB_recO_recG-2629_wt | 0 |
| 3604_recO_ruvABC_recG-2629_wt | 0 |
| 3605_recB_recO_ruvABC_recG-2629_wt | 0 |
| 3610_ruvABC_recG-2629_wt | 0 |
| 3625_recB_ruvABC-2629_wt | 4,3616E-10 |
| 3626_recB_recG-2629_wt | 0 |
| 4134_recB_ruvABC_recG-2629_wt | 0 |
| 3188_ruvABC-3183_recB | 0,69726016 |
| 3196_recG-3183_recB | 0,00027346 |
| 3545_recO-3183_recB | 0,03584529 |
| 3599__recB_recO-3183_recB | 0 |
| 3600_recO_ruvABC-3183_recB | 0,24161365 |
| 3601_recO_recG-3183_recB | 0,99995333 |
| 3602_recB_recO_ruvABC-3183_recB | 7,415E-12 |
| 3603_recB_recO_recG-3183_recB | 0 |
| 3604_recO_ruvABC_recG-3183_recB | 0 |
| 3605_recB_recO_ruvABC_recG-3183_recB | 0 |
| 3610_ruvABC_recG-3183_recB | 0 |
| 3625_recB_ruvABC-3183_recB | 0,33564124 |
| 3626_recB_recG-3183_recB | 1,6997E-06 |
| 4134_recB_ruvABC_recG-3183_recB | 1,1737E-10 |
| 3196_recG-3188_ruvABC | 0,30219522 |
| 3545_recO-3188_ruvABC | 0,98777929 |
| 3599__recB_recO-3188_ruvABC | 0 |
| 3600_recO_ruvABC-3188_ruvABC | 0,00034155 |
| 3601_recO_recG-3188_ruvABC | 0,99171242 |
| 3602_recB_recO_ruvABC-3188_ruvABC | 0 |
| 3603_recB_recO_recG-3188_ruvABC | 0 |
| 3604_recO_ruvABC_recG-3188_ruvABC | 0 |
| 3605_recB_recO_ruvABC_recG-3188_ruvABC | 0 |
| 3610_ruvABC_recG-3188_ruvABC | 0 |
| 3625_recB_ruvABC-3188_ruvABC | 1 |
| 3626_recB_recG-3188_ruvABC | 4,0045E-10 |
| 4134_recB_ruvABC_recG-3188_ruvABC | 0 |
| 3545_recO-3196_recG | 0,98638171 |
| 3599__recB_recO-3196_recG | 0 |
| 3600_recO_ruvABC-3196_recG | 3,6165E-10 |
| 3601_recO_recG-3196_recG | 0,00625197 |

| | |
|---|---|
| 3602_recB_recO_ruvABC-3196_recG | 0 |
| 3603_recB_recO_recG-3196_recG | 0 |
| 3604_recO_ruvABC_recG-3196_recG | 0 |
| 3605_recB_recO_ruvABC_recG-3196_recG | 0 |
| 3610_ruvABC_recG-3196_recG | 0 |
| 3625_recB_ruvABC-3196_recG | 0,24265865 |
| 3626_recB_recG-3196_recG | 0 |
| 4134_recB_ruvABC_recG-3196_recG | 0 |
| 3599__recB_recO-3545_recO | 0 |
| 3600_recO_ruvABC-3545_recO | 4,5736E-07 |
| 3601_recO_recG-3545_recO | 0,28538958 |
| 3602_recB_recO_ruvABC-3545_recO | 0 |
| 3603_recB_recO_recG-3545_recO | 0 |
| 3604_recO_ruvABC_recG-3545_recO | 0 |
| 3605_recB_recO_ruvABC_recG-3545_recO | 0 |
| 3610_ruvABC_recG-3545_recO | 0 |
| 3625_recB_ruvABC-3545_recO | 0,99504396 |
| 3626_recB_recG-3545_recO | 0 |
| 4134_recB_ruvABC_recG-3545_recO | 0 |
| 3600_recO_ruvABC-3599__recB_recO | 1,9866E-10 |
| 3601_recO_recG-3599__recB_recO | 0 |
| 3602_recB_recO_ruvABC-3599__recB_recO | 0,99045409 |
| 3603_recB_recO_recG-3599__recB_recO | 0,89271856 |
| 3604_recO_ruvABC_recG-3599__recB_recO | 7,0965E-05 |
| 3605_recB_recO_ruvABC_recG-3599__recB_recO | 0,99871845 |
| 3610_ruvABC_recG-3599__recB_recO | 0,00284946 |
| 3625_recB_ruvABC-3599__recB_recO | 0 |
| 3626_recB_recG-3599__recB_recO | 4,0627E-05 |
| 4134_recB_ruvABC_recG-3599__recB_recO | 0,98806236 |
| 3601_recO_recG-3600_recO_ruvABC | 0,02818402 |
| 3602_recB_recO_ruvABC-3600_recO_ruvABC | 1,9294E-08 |
| 3603_recB_recO_recG-3600_recO_ruvABC | 3,6675E-12 |
| 3604_recO_ruvABC_recG-3600_recO_ruvABC | 0 |
| 3605_recB_recO_ruvABC_recG-3600_recO_ruvABC | 4,9474E-11 |
| 3610_ruvABC_recG-3600_recO_ruvABC | 0 |
| 3625_recB_ruvABC-3600_recO_ruvABC | 9,7852E-06 |
| 3626_recB_recG-3600_recO_ruvABC | 0,01335123 |
| 4134_recB_ruvABC_recG-3600_recO_ruvABC | 2,4178E-07 |
| 3602_recB_recO_ruvABC-3601_recO_recG | 0 |
| 3603_recB_recO_recG-3601_recO_recG | 0 |
| 3604_recO_ruvABC_recG-3601_recO_recG | 0 |
| 3605_recB_recO_ruvABC_recG-3601_recO_recG | 0 |
| 3610_ruvABC_recG-3601_recO_recG | 0 |
| 3625_recB_ruvABC-3601_recO_recG | 0,90643227 |
| 3626_recB_recG-3601_recO_recG | 6,4028E-08 |
| 4134_recB_ruvABC_recG-3601_recO_recG | 6,0801E-12 |
| 3603_recB_recO_recG-3602_recB_recO_ruvABC | 0,1722987 |
| 3604_recO_ruvABC_recG-3602_recB_recO_ruvABC | 9,2279E-07 |
| 3605_recB_recO_ruvABC_recG-3602_recB_recO_ruvABC | 0,56713899 |
| 3610_ruvABC_recG-3602_recB_recO_ruvABC | 7,4233E-05 |

| | |
|---|---|
| 3625_recB_ruvABC-3602_recB_recO_ruvABC | 0 |
| 3626_recB_recG-3602_recB_recO_ruvABC | 0,00448488 |
| 4134_recB_ruvABC_recG-3602_recB_recO_ruvABC | 1 |
| 3604_recO_ruvABC_recG-3603_recB_recO_recG | 0,04529139 |
| 3605_recB_recO_ruvABC_recG-3603_recB_recO_recG | 0,99999822 |
| 3610_ruvABC_recG-3603_recB_recO_recG | 0,28138471 |
| 3625_recB_ruvABC-3603_recB_recO_recG | 0 |
| 3626_recB_recG-3603_recB_recO_recG | 2,8975E-07 |
| 4134_recB_ruvABC_recG-3603_recB_recO_recG | 0,20109602 |
| 3605_recB_recO_ruvABC_recG-3604_recO_ruvABC_recG | 0,00702183 |
| 3610_ruvABC_recG-3604_recO_ruvABC_recG | 1 |
| 3625_recB_ruvABC-3604_recO_ruvABC_recG | 0 |
| 3626_recB_recG-3604_recO_ruvABC_recG | 0 |
| 4134_recB_ruvABC_recG-3604_recO_ruvABC_recG | 2,8617E-06 |
| 3610_ruvABC_recG-3605_recB_recO_ruvABC_recG | 0,07894857 |
| 3625_recB_ruvABC-3605_recB_recO_ruvABC_recG | 0 |
| 3626_recB_recG-3605_recB_recO_ruvABC_recG | 3,7393E-06 |
| 4134_recB_ruvABC_recG-3605_recB_recO_ruvABC_recG | 0,58713941 |
| 3625_recB_ruvABC-3610_ruvABC_recG | 0 |
| 3626_recB_recG-3610_ruvABC_recG | 1,8721E-10 |
| 4134_recB_ruvABC_recG-3610_ruvABC_recG | 0,0001372 |
| 3626_recB_recG-3625_recB_ruvABC | 0 |
| 4134_recB_ruvABC_recG-3625_recB_ruvABC | 0 |
| 4134_recB_ruvABC_recG-3626_recB_recG | 0,01974773 |
